# Psychometric properties and meaningful change thresholds for the QOL-E instrument in patients with myelodysplastic neoplasms

**DOI:** 10.3389/fonc.2025.1507854

**Published:** 2025-02-07

**Authors:** Esther Natalie Oliva, Shien Guo, Jennifer Lord-Bessen, Aylin Yucel, Roberto Latagliata, Massimo Breccia, Giuseppe A. Palumbo, Grazia Sanpaolo, Marta Riva, Valeria Santini, Uwe Platzbecker, Guillermo Garcia-Manero, Pierre Fenaux, Christopher G. Pelligra

**Affiliations:** ^1^ Hematology Unit, Grande Ospedale Metropolitano Bianchi Melacrino Morelli, Reggio Calabria, Italy; ^2^ Evidera, Waltham, MA, United States; ^3^ Bristol Myers Squibb, Lawrence, NJ, United States; ^4^ Hematology Unit, Belcolle Hospital, Viterbo, Italy; ^5^ Hematology, Department of Translational and Precision Medicine, Az. Policlinico Umberto I-Sapienza University, Rome, Italy; ^6^ Department of Medical, Surgical Sciences and Advanced Technologies “GF Ingrassia”, University of Catania, Catania, Italy; ^7^ Department of Hematology and Stem Cell Transplantation Unit, IRCCS Casa Sollievo della Sofferenza Hospital, San Giovanni Rotondo, Italy; ^8^ Department of Hematology and Oncology, Niguarda Cancer Center, ASST Grande Ospedale Metropolitano Niguarda, Milan, Italy; ^9^ MDS Unit, Hematology, DMSC, University of Florence, AOUC, Florence, Italy; ^10^ Medical Clinic and Policlinic 1, Hematology and Cellular Therapy, University Hospital Leipzig, Leipzig, Germany; ^11^ Department of Leukemia, University of Texas MD Anderson Cancer Center, Houston, TX, United States; ^12^ Service d’Hématologie Séniors, Hôpital Saint-Louis, Université Paris 7, Paris, France; ^13^ Evidera, Atlanta, GA, United States

**Keywords:** health-related quality of life, myelodysplastic syndromes, myelodysplastic neoplasms, patient-reported outcomes, psychometric analysis, QOL-E, clinically meaningful changes

## Abstract

**Background:**

Myelodysplastic neoplasms (MDS) are characterized by ineffective hematopoiesis, peripheral blood cytopenias, and an increased risk of progression to acute myeloid leukemia. One of the main treatment goals is improving quality of life (QoL), particularly for patients with lower-risk MDS (LR-MDS) who may live longer with compromised QoL. The QOL-E^©^ is a patient-reported outcome (PRO) measure specifically developed to address the lack of a health-related QoL questionnaire for patients with MDS. The objective of this study was to evaluate the psychometric performance of the QOL-E in patients with LR-MDS.

**Methods:**

Data from four clinical trials in MDS (MEDALIST, DARB-MDS, EQoL-MDS, and RevMDS trials) were used to assess construct validity, reliability, and responsiveness. The QOL-E was validated by the European Organization for the Research and Treatment of Cancer (EORTC) Quality of Life Questionnaire – Core 30 (QLQ-C30) and clinical outcomes. It contains 29 items with the first two items assessing the patient’s general well-being and the 27 remaining items grouped into six domain scores: physical well-being (QOL-FIS), functional well-being (QOL-FUN), social/family well-being (QOL-SOC), sexual well-being (QOL-SEX), fatigue (QOL-FAT), and MDS-specific disturbances (QOL-MDSS). Additionally, meaningful within-patient change (MWPC) thresholds were determined for the domains and summary scores of the QOL-E using anchor-based analyses, supported by distribution-based analyses.

**Results:**

A total of 458 patients were included in the analyses. The QOL-E domain/summary scores demonstrated acceptable convergent/divergent and known-groups validity. Test-retest reliability and internal consistency was confirmed with intraclass correlation coefficients and Cronbach alpha exceeding 0.70 across most QOL-E domains/summary scores. The QOL-E domains/summary scores, except for QOL-SEX, had an adequate ability to detect change from baseline to Week 24. MWPC thresholds were proposed for all other domains and summary scores.

**Conclusion:**

The study results demonstrate that the QOL-E is generally fit for purpose to assess treatment effects in populations with LR-MDS and the proposed MWPC thresholds can be used to assess within-patient treatment effect on PROs, as assessed by the QOL-E, in future studies.

## Introduction

Myelodysplastic neoplasms (MDS) are characterized by ineffective hematopoiesis resulting in peripheral blood cytopenias and increased risk of progression to acute myeloid leukemia (AML) (1, 2). Patients may be categorized into five risk groups (Very low-, Low-, Intermediate-, High-, and Very high-risk), according to the Revised International Prognostic Scoring System (IPSS-R), based on cytogenetic features, marrow blast percentage, and depth of cytopenia (3). Patients with lower-risk MDS (LR-MDS) typically present with severe and chronic anemia leading to increased morbidity as a result of anemia-related symptoms such as fatigue and an increased risk of cardiac complications; all of which can have profound impacts on their life expectancy and quality of life (QoL) (4–7).

Apart from allogeneic hematopoietic stem cell transplantation, which is not suitable for most patients due to advanced age and/or comorbidities, current treatment options are not curative (8, 9). Instead, the main treatment goals are to improve or eliminate cytopenias for patients with lower-risk disease, to prevent or slow progression to AML for higher-risk patients, and to maintain or improve QoL for all patients (10, 11).

Red blood cell (RBC) transfusions are commonly employed as a form of supportive care to alleviate symptoms associated with anemia. They can offer temporary relief or prevent symptoms from worsening (12–14). Nevertheless, relying on RBC transfusions over the long term can lead to complications such as excessive iron accumulation (which may cause cardiac and hepatic organ failure) or immune-related disorders (12, 15, 16). Regular RBC transfusions and related complications have the potential to significantly impact various aspects of a patient’s QoL, including their social (e.g., missing work, decreased social interactions) and emotional well-being (e.g., anxiety/depression, fatigue) (7, 17). Indeed, patients with LR-MDS have reported poorer health-related QoL (HRQoL) compared with the general population (7).

The burden of MDS and its treatments on HRQoL emphasizes the importance of patient-reported outcome (PRO) instruments which can measure concepts relevant and specific to patients with MDS. Disease-specific PROs are essential to evaluate the impact of disease and treatment on HRQoL both in clinical practice and in research, particularly for patients with LR-MDS who may live longer with compromised HRQoL (18). The QOL-E^©^ is a questionnaire that was developed to assess disease-specific issues and aspects of overall well-being for patients with MDS (19). Its development was based on concept elicitation via a patient focus group, followed by a pilot study including cognitive debriefing and field testing where the instrument was administered to 147 patients for a preliminary evaluation of psychometric performance (19).

The QOL-E questionnaire has been used in several clinical trials to assess HRQoL in patients with LR-MDS. The phase 3 MEDALIST trial compared treatment with luspatercept + best supportive care (BSC) to placebo + BSC in patients with transfusion-dependent anemia due to LR-MDS (20). No clinically meaningful differences were found in all QOL-E domains between and within the two groups through Week 25, suggesting that luspatercept treatment maintained patients’ QoL levels while reducing RBC transfusion burden (21). One single item of the QOL-E questionnaire, specifically related to transfusion dependence, showed improvement in daily life owing to reduction of transfusion burden in the luspatercept treatment arm versus placebo. In the phase 2 DARB-MDS study, the efficacy, safety, and changes in biological features of hematopoietic progenitors and QoL associated with darbepoetin alfa treatment were evaluated in patients with International Prognostic Scoring System (IPSS)-defined Low and Intermediate-1 risk MDS (22). For QOL-E, mean total scores significantly improved through Week 24. Hemoglobin (Hb) increases were linked to improvements in physical (QOL-FIS), functional (QOL-FUN), and social/family (QOL-SOC) well-being, and general (QOL-GEN) QOL-E domains, particularly in the first 8 weeks. The phase 2 EQoL-MDS study compared eltrombopag with placebo in patients with LR-MDS and severe persistent thrombocytopenia (23, 24). No significant changes were observed in QOL-E items within or between the two groups. However, improvements in QOL-E QOL-SOC, sexual well-being (QOL-SEX), MDS-specific disturbances (QOL-MDSS), treatment outcome index (QOL-TOI), and QOL-GEN scores were noted with increasing platelet counts. The RevMDS study evaluated the efficacy, safety, and HRQoL changes associated with lenalidomide treatment in patients with anemia and Low and Intermediate-1 risk MDS with del(5q), with or without additional cytogenetic abnormalities (25). Lenalidomide was associated with clinically meaningful improvements in HRQoL. Significant improvements were seen in the QOL-E QOL-FIS and QOL-SOC domains at Week 8 and Week 24, respectively, with benefits sustained through 52 weeks. Of note, patients with poor baseline HRQoL (those considered in need of treatment) showed improvements across QOL-FIS, QOL-FUN, QOL-SOC, and QOL-TOI.

Despite its use in several clinical trials, only content and construct validity, and reliability have been established for the QOL-E (19). Its convergent/divergent validity, known-groups validity, responsiveness, and score interpretability have yet to be established. These measurement properties are included in guidance from the United States (US) Food and Drug Administration (FDA) for the use of PROs related to medical treatments (26–28). The purpose of this study was to further evaluate the psychometric performance of the QOL-E for assessing HRQoL in patients with LR-MDS and to determine the thresholds for defining meaningful within-patient changes (MWPCs) in QOL-E domain and summary scores.

## Materials and methods

### Study design and outcome assessment

The psychometric evaluation used data from four clinical trials in MDS for which patient-level data were available: MEDALIST (21), DARB-MDS (22), EQoL-MDS (23, 24), and RevMDS (25). The key study characteristics are summarized in **Supplementary Table 1**. The studies included patients with Very low-, Low-, or Intermediate-risk MDS on the IPSS-R (MEDALIST) (21) or Low- or Intermediate-1-risk MDS on the IPSS (DARB-MDS, EQoL-MDS, and RevMDS) (22–25). The PRO assessment time points differed among the four studies, but all studies administered the QOL-E at baseline and Week 24 (i.e., within 4 weeks of Day 168 from baseline). The European Organization for the Research and Treatment of Cancer (EORTC) Quality of Life Questionnaire – Core 30 (QLQ-C30), which was administered in all studies except the RevMDS study, was also used to validate the QOL-E. Clinical outcomes used to validate the QOL-E included: Hb level, RBC units transfused in the previous 8 weeks, platelet count, and platelet units transfused in the previous 8 weeks; only values collected at both baseline and Week 24 were considered in the analysis.

The QOL-E (version 3) is a 29-item questionnaire, with the first two items assessing a patient’s general well-being relative to a month prior. The remaining 27 items form six domain scores: QOL-FIS, QOL-FUN, QOL-SOC, QOL-SEX, fatigue (QOL-FAT), and QOL-MDSS. The recall periods for each item of the six QOL-E domain scores are shown in **Supplementary Table 2**. Three summary scores are derived from the domain scores: QOL-GEN, calculated by taking the mean of all domains except for QOL-MDSS; QOL-ALL, calculated by taking the mean of QOL-GEN and QOL-MDSS; and the QOL-TOI, calculated by taking the mean of QOL-FIS, QOL-FUN, and QOL-MDSS. All domain and summary scores were standardized into a scale ranging from 0 (worst outcome) to 100 (best outcome). Further information related to scoring the QOL-E can be found in the **Supplementary Material**. Of note, version 3 (various languages) of the QOL-E was used in the MEDALIST and EQoL-MDS studies, while version 2 (Italian language) of the QOL-E was used in the DARB-MDS and RevMDS studies (**Supplementary Table 1**); however, only minor wording changes were made between the two versions, which were deemed unlikely to cause any difference in patient responses. Specifically, in the Italian version, Item 7 “Your health is an impediment for you to keep a paid job (whether you are of retirement age or not)” was reworded to be more comprehensive from previous versions after linguistic translations and cognitive interviews were conducted. At the time of this writing, the questionnaire has been translated and linguistically validated in 27 languages across 18 countries and is available at https://qol-e.it/questionnaire/.

### Statistical analyses

The analysis population included all patients from the four studies with a non-missing baseline QOL-E domain score, unless otherwise noted below. Data from patients participating in the RevMDS study were excluded from any analyses including the EORTC QLQ-C30 since the EORTC QLQ-C30 was not administered in that study. Analyses focused on the common time points among all four studies, baseline and Week 24, unless otherwise specified. Additionally, where it was possible to do so, pooled results from these two time points were reported.

#### Psychometric validation

##### Distributional properties

To assess the floor effects, ceiling effects, and score variability of the QOL-E, tabulations of the numbers and percentage of patients falling into each of the ten-point incremental categories at baseline and Week 24 were summarized for each domain and summary score. Additionally, descriptive statistics (mean, standard deviation [SD], median, first and third quartiles [Q1 and Q3], minimum, and maximum) were calculated. A problematic floor or ceiling effect were considered to be present if more than 15% of patients had a score of 0 or 100, respectively (29–31).

##### Construct validity

Convergent and divergent validity evaluate the degree to which a scale under evaluation relates to others with which it is and is not, respectively, expected to be related (32). In this analysis, convergent and divergent validity were assessed by estimating the correlation between the QOL-E domains/summary scores and scores or measurements from other outcomes measuring similar or different concepts and comparing the correlations to hypotheses prespecified in the statistical analysis plan. Spearman rank correlation coefficients (and corresponding P values) between QOL-E domain/summary scores, EORTC QLQ-C30 domain scores, and the selected clinical outcomes (Hb level, RBC units transfused in the previous 8 weeks, platelet count, and platelet units transfused in the previous 8 weeks) were estimated.

The sensitivity of the QOL-E to differentiate specific groups of patients known to be different in a relevant way (i.e., known-groups validity) was also assessed by comparing distributions of scores (i.e., median, Q1, and Q3) of each QOL-E domain and summary score among the following known groups: (i) baseline response on the QOL-E Item 1 “In general, you would say that your health is: excellent, good, acceptable, or poor” and (ii) baseline RBC transfusion dependency (patients who were transfusion dependent at baseline received ≥1 RBC units in the previous 8 weeks and patients who were non-transfusion dependent at baseline received 0 RBC units in the previous 8 weeks; yes, no).

##### Reliability

Test-retest reliability, measuring the extent to which a measure yields consistent scores within the same participants each time it is administered over a short period of time, was assessed via the intraclass correlation coefficient (ICC) among stable patients (i.e., patients reporting the same overall health status between two different time points). This analysis was performed on a subset of the analysis population which included only those who participated in the MEDALIST study, as this was the only study to evaluate the QOL-E at two time points, which were close together (i.e., at a screening visit between 14 and 35 days prior to baseline and at baseline). Item 1 of the QOL-E (“In general, you would say that your health is: excellent, good, acceptable, or poor”) was used to define stable patients; those reporting the same response at both time points were considered to be “stable.” The ICC for the test-retest reliability of each domain or summary score was calculated using a two-way mixed-effect analysis of variance (ANOVA) model with interaction for the absolute agreement between single scores (i.e., ICC (A,1) in the McGraw and Wong naming convention) (33, 34). ICC values ≥0.70 were regarded as an acceptable range for the test-retest reliability (32).

Internal consistency reflects how items or subscales comprising an instrument measure the same underlying construct (35). Cronbach alpha was used to assess the degree of internal consistency of responses to the items within each of the QOL-E domains (36). Additionally, omega coefficients were also estimated for all domains and summary scores (i.e., QOL-GEN, QOL-ALL, and QOL-TOI), eliminating the need for the assumption of tau-equivalence (i.e., that all items comprising the scale contribute equally on the same scale and measure the same inherent variable) assumed by Cronbach alpha (37–39). Values of standardized alpha coefficients after deletion of individual items were also presented for each domain score of the QOL-E. Standardized alpha coefficients or omega coefficients ≥0.70 were regarded as demonstrating acceptable/good internal consistency (40). Additionally, to support internal consistency analyses, inter-domain correlations (Spearman) and correlations between domains and the corrected summary score (i.e., the summary score in question calculated excluding the domain in question) were estimated.

##### Responsiveness (sensitivity to change)

The sensitivity of the QOL-E domain and summary scores to respond to change in concepts of interest was evaluated by estimating the correlation (Spearman) of changes in each of the QOL-E domains/summary scores from baseline to Week 24 with changes in the selected external anchors (**Supplementary Table 3**) over the same time period.

#### Determination of MWPC thresholds

All analyses were conducted in accordance with US FDA draft guidance (26–28). MWPC thresholds (i.e., the responder definitions) were estimated primarily from an anchor-based approach, supported by estimates from a distribution-based approach. Patients were categorized based on levels of change in a given anchor (hereafter referred to as “anchor group”) at Week 24 from baseline. Mean and median score estimates from a given anchor group and estimates from the distribution-based analyses were triangulated to determine the MWPC threshold for each QOL-E domain/summary score.

##### Anchor selection

Multiple potential anchors were explored to provide cumulative evidence to help interpretation. The list of potential anchors included the same measures used in the correlational responsiveness analysis, as described in **Supplementary Table 3**. The QOL-E Items 1 and 2, which are not included in any of the QOL-E domain or summary scores, were considered as potential anchors as they ask about patients’ overall health (or change in health) using verbal rating scales that can be easily interpreted. The EORTC QLQ-C30 Items 29 and 30 were also considered as potential anchors as they are also plainly understood self-reported measures asking about health and QoL. Finally, Hb and RBC transfusion burden levels were also examined because they are important clinical outcomes for patients with MDS, especially those requiring RBC transfusions. Among the potential anchors, those with a correlation coefficient exceeding 0.3 in absolute value across the QOL-E domains and summary scores were included in the anchor-based analysis.

##### Anchor-based analyses

Patients were categorized according to each anchor group based on their level of change on the chosen anchors (as defined in **Supplementary Table 3**). Descriptive statistics and empirical cumulative distribution function (eCDF) curves for the change from baseline in QOL-E scores were produced for each anchor group. If any anchor group had a sample size of less than or equal to ten patients, they were collapsed with the adjacent anchor group. Descriptive statistics of observed change from baseline (number of patients, median, mean) in each of the QOL-E domains and summary scores at Week 24 were summarized for each anchor group.

##### Distribution-based analyses

A distribution-based analysis was conducted to support the selection of the thresholds for MWPCs. This analysis was performed on a subset of the analysis population which included only those who participated in the MEDALIST study because this was the only trial whose study design allowed a calculation of ICC. Two estimates were used: 1) ± 1 standard error of measurement (SEM; taken as the baseline SD of the QOL-E score multiplied by $\sqrt{1-ICC}$ ), which is typically considered as the lower bound of meaningful threshold as it estimates the amount of measurement error associated with the measure, and 2) half of the SD of the QOL-E domain/summary score at baseline (i.e., corresponding to an effect size of 0.5). Both have been suggested to represent a clinically important difference (41, 42).

##### Triangulation

To identify the MWPC thresholds, the first step was to determine which level(s) of improvement (or worsening) on an anchor could be used to represent a meaningful improvement (or worsening) among the target population in the context of the study. To determine this, the eCDF plots were examined. If the curves between the groups with ≥1 level of improvement (or ≥1 level of worsening) and no change were clearly and consistently separated, then the estimates (i.e., mean and median change from baseline) from the group with ≥1 level of improvement (or worsening) were considered in the triangulation for each domain or summary score. An MWPC threshold for each direction (improvement/deterioration) was then proposed from the range of the anchor-based estimates by considering possible state changes of the target domain (i.e., the minimum possible change in each standardized 0–100 domain score that an individual patient could experience) and the lower bound threshold set by SEM for that domain (i.e., MWPC threshold should be ≥SEM).

## Results

### Demographics and baseline characteristics

A total of 458 patients were included in the analyses (227 [49.6%] from MEDALIST, 34 [7.4%] from DARB-MDS, 158 [34.5%] from EQoL-MDS, and 39 [8.5%] from RevMDS). Baseline demographic and disease characteristics are reported in **Table 1**. The majority of patients had LR-MDS (i.e., IPSS-R score of Very low, Low, or Intermediate; 95.4%) and were transfusion dependent (68.3%); median time since the initial diagnosis of MDS was 29.2 months.

**Table 1:** Demographics and baseline characteristics.

Characteristic/statistic	Analysis population (N=458)
Age (years)	
Mean (SD)	70.5 (10.4)
Median	72.0
Min, Max	26.0, 95.0
Sex, n (%)	
Male	268 (58.5%)
Female	190 (41.5%)
Race, n (%)	
Asian	2 (0.4%)
Black or African American	1 (0.2%)
White	385 (84.0%)
Other	2 (0.4%)
Not collected or reported	68 (14.9%)
Time since initial diagnosis of MDS (months)	
Mean (SD)	41.6 (44.7)
Median	29.2
Min, Max	0.0, 420.6
IPSS-R risk, n (%)^a^	
Very low	30 (6.6%)
Low	310 (67.7%)
Intermediate	97 (21.2%)
High	20 (4.4%)
Very high	1 (0.2%)
Hb level (g/dL)	
N	404
Mean (SD)	9.5 (1.9)
Median	9.1
Min, Max	5.2, 16.6
Platelet count, n (%)	
<100×10^9^/L	170 (37.1%)
≥100-≤400×10^9^/L	185 (40.4%)
>400×10^9^/L	49 (10.7%)
RBC transfusion-dependent, n (%)	
Yes	313 (68.3%)
No	131 (28.6%)

Hb, hemoglobin; IPSS, International Prognostic Scoring System; IPSS-R, Revised IPSS; Max, maximum; MDS, myelodysplastic neoplasms; Min, minimum; RBC, red blood cell; SD, standard deviation.

a For the DARB-MDS, EQoL-MDS, and RevMDS studies, IPSS was converted to IPSS-R based on clinical expert consultation.

Baseline QOL-E scores are summarized in **Supplementary Table 4**. Mean QOL-E scores ranged from 47.5 (QOL-SOC) to 74.0 (QOL-FAT). No problematic floor effects (i.e., more than 15% of patients [excluding missing] with a score of 0) were noted except for the QOL-SOC, indicating a large proportion (25.1%, excluding missing) of patients experienced maximum impacts on social and family life at baseline. Problematic ceiling effects (i.e., more than 15% of patients [excluding missing] with a score of 100) were observed for the QOL-FUN, QOL-SOC, and QOL-SEX domains, indicating a large proportion of patients experiencing no impact on functional well-being, social/family life, and sexual well-being (25.0%, 20.4%, and 37.4%, respectively [excluding missing]).

### Psychometric validation

#### Construct validity

The directions and magnitudes of the Spearman rank correlations between the QOL-E domain scores and the QLQ-C30 domain scores pooled across baseline and Week 24 (**Table 2**) were generally consistent with *a priori* hypotheses; the exceptions included the QOL-SEX domain, which showed a weak correlation (|r|<0.3) with pain rather than the hypothesized moderate correlation (0.3 ≤ |r|<0.7), and the QOL-MDSS domain, which showed a moderate correlation with social functioning rather than the hypothesized weak correlation. In some cases, the QOL-E summary scores showed higher correlations with the QLQ-C30 domain scores than hypothesized, particularly for the functioning domains.

**Table 2:** Convergent and divergent validity: Spearman correlations between QOL-E domain and summary scores and other outcome measures.

Measure	QOL-FIS n r P value	QOL-FUN n r P value	QOL-SOC n r P value	QOL-SEX n r P value	QOL-FAT n r P value	QOL-MDSS n r P value	QOL-GEN n r P value	QOL-ALL n r P value	QOL-TOI n r P value
EORTC QLQ-C30									
Global Health Status/QoL	622 0.43 <0.001	615 0.49 <0.001	553 0.53 <0.001	547 0.20 <0.001	626 0.56 <0.001	618 0.51 <0.001	540 0.62 <0.001	537 0.62 <0.001	602 0.59 <0.001
Physical functioning	624 0.67 <0.001	617 0.56 <0.001	555 0.57 <0.001	549 0.11 0.009	628 0.60 <0.001	620 0.57 <0.001	542 0.67 <0.001	539 0.68 <0.001	604 0.73 <0.001
Role functioning	622 0.55 <0.001	615 0.62 <0.001	554 0.59 <0.001	549 0.21 <0.001	626 0.63 <0.001	619 0.60 <0.001	541 0.71 <0.001	538 0.72 <0.001	603 0.73 <0.001
Emotional functioning	621 0.35 <0.001	615 0.41 <0.001	553 0.54 <0.001	547 0.22 <0.001	625 0.51 <0.001	618 0.49 <0.001	540 0.56 <0.001	537 0.57 <0.001	602 0.50 <0.001
Cognitive functioning	622 0.31 <0.001	615 0.40 <0.001	553 0.42 <0.001	548 0.19 <0.001	626 0.45 <0.001	618 0.40 <0.001	540 0.47 <0.001	537 0.46 <0.001	602 0.44 <0.001
Social functioning	620 0.42 <0.001	613 0.49 <0.001	552 0.59 <0.001	548 0.26 <0.001	623 0.51 <0.001	617 0.61 <0.001	540 0.62 <0.001	537 0.67 <0.001	602 0.62 <0.001
Fatigue	621 −0.51 <0.001	614 −0.63 <0.001	553 −0.57 <0.001	548 −0.19 <0.001	625 −0.68 <0.001	618 −0.57 <0.001	540 −0.69 <0.001	537 −0.69 <0.001	602 −0.70 <0.001
Nausea and vomiting	623 −0.21 <0.001	616 −0.26 <0.001	555 −0.22 <0.001	549 0.00 0.996	627 −0.34 <0.001	620 −0.21 <0.001	542 −0.26 <0.001	539 −0.25 <0.001	604 −0.27 <0.001
Pain	623 −0.36 <0.001	616 −0.37 <0.001	554 −0.40 <0.001	548 −0.16 <0.001	627 −0.47 <0.001	619 −0.36 <0.001	541 −0.47 <0.001	538 −0.45 <0.001	603 −0.44 <0.001
Dyspnea	619 −0.38 <0.001	612 −0.47 <0.001	550 −0.35 <0.001	544 −0.17 <0.001	622 −0.48 <0.001	614 −0.40 <0.001	538 −0.50 <0.001	535 −0.49 <0.001	599 −0.51 <0.001
Insomnia	619 −0.26 <0.001	612 −0.28 <0.001	552 −0.30 <0.001	547 −0.08 0.074	623 −0.45 <0.001	617 −0.29 <0.001	539 −0.33 <0.001	536 −0.34 <0.001	601 −0.32 <0.001
Appetite loss	621 −0.31 <0.001	614 −0.35 <0.001	553 −0.30 <0.001	547 0.01 0.880	625 −0.41 <0.001	618 −0.29 <0.001	540 −0.36 <0.001	537 −0.35 <0.001	602 −0.37 <0.001
Constipation	623 −0.18 <0.001	616 −0.19 <0.001	554 −0.22 <0.001	548 −0.03 0.426	627 −0.29 <0.001	619 −0.20 <0.001	541 −0.24 <0.001	538 −0.25 <0.001	603 −0.22 <0.001
Diarrhea	621 −0.14 <0.001	615 −0.12 0.004	552 −0.09 0.036	547 −0.06 0.138	625 −0.10 0.010	617 −0.10 0.014	540 −0.12 0.004	537 −0.11 0.008	602 −0.15 <0.001
Financial difficulties	615 −0.23 <0.001	608 −0.18 <0.001	548 −0.30 <0.001	546 −0.21 <0.001	618 −0.30 <0.001	613 −0.30 <0.001	536 −0.32 <0.001	533 −0.34 <0.001	598 −0.28 <0.0001
Clinical outcomes									
Hb level	623 0.26 <0.001	618 0.18 <0.001	556 0.18 <0.001	518 0.04 0.352	629 0.15 <0.001	618 0.20 <0.001	542 0.23 <0.001	535 0.23 <0.001	599 0.24 <0.001
RBC units transfused in the last 8 weeks	683 −0.17 <0.001	678 −0.18 <0.001	611 −0.15 <0.001	575 −0.08 0.042	689 −0.11 0.006	677 −0.24 <0.001	597 −0.21 <0.001	590 −0.24 <0.001	658 −0.24 <0.001
Platelet count	623 −0.06 0.152	618 −0.01 0.882	556 −0.01 0.819	518 0.00 0.981	629 −0.02 0.654	618 −0.02 0.578	542 −0.01 0.873	535 −0.01 0.762	599 −0.02 0.678
Platelet units transfused in the last 8 weeks	682 −0.07 0.062	677 −0.10 0.013	610 −0.13 0.001	575 0.04 0.384	688 −0.07 0.059	676 −0.13 0.001	596 −0.12 0.003	589 −0.13 0.001	657 −0.13 0.001

EORTC, European Organization for Research and Treatment of Cancer; Hb, hemoglobin; QLQ-C30, Quality of Life Questionnaire-Core 30; QoL, quality of life; QOL-ALL, calculated by taking the mean of QOL-GEN and QOL-MDSS; QOL-FAT, fatigue; QOL-FIS, physical well-being; QOL-FUN, functional well-being; QOL-GEN, calculated by taking the mean of all domains except for QOL-MDSS; QOL-SEX, sexual well-being; QOL-SOC, social/family well-being; QOL-MDSS, MDS-specific disturbances; QOL-TOI, treatment outcome index calculated by taking the mean of QOL-FIS, QOL-FUN, and QOL-MDSS; RBC, red blood cell.

Correlations are pooled across both baseline and Week 24. Data from patients participating in the RevMDS study were excluded from the EORTC QLQ-C30 correlations as the EORTC QLQ-C30 was not administered in that study. n indicates the number of observations included in the analysis (may include up to two observations, at baseline and Week 24, from a single patient), r is the correlation coefficient, and P is the P value testing a non-zero correlation. Cells in dark gray indicate a weaker correlation than hypothesized, cells in light gray indicate a stronger correlation than hypothesized, and cells in white indicate correlations were as hypothesized. All correlations were in the direction hypothesized or had P value >0.05 indicating no significant correlation in either direction.

QOL-E domains/summary scores showed weak correlations with all clinical outcomes investigated (i.e., Hb level, RBC units transfused in the previous 8 weeks, platelet count, and platelet units transfused in the previous 8 weeks), which was mostly consistent with *a priori* hypotheses. Overall, the results showed that the QOL-E domains and summary scores have adequate convergent and divergent validity.

When assessing known groups based on patients’ overall health status (i.e., excellent, good, acceptable, and poor) as captured by the QOL-E Item 1 “In general, you would say that your health is: excellent, good, acceptable, or poor” (**Table 3**), median scores for all QOL-E domains/summary scores were clearly different between these four groups. However, for the QOL-SEX domain, interquartile ranges (i.e., Q1 to Q3) overlapped among all four groups (i.e., excellent, good, acceptable, and poor), indicating that the domain was less able to differentiate among the groups than the other domains and summary scores. When defining known groups by transfusion dependency (i.e., RBC transfusion dependent and RBC transfusion independent; **Supplementary Table 5**), the RBC transfusion-dependent group tended to have slightly worse scores than the transfusion-independent group, as expected, although the differentiation between groups was less than that when groups were defined by overall health status. Overall, the findings indicate that most QOL-E domains and summary scores were able to differentiate between subgroups of patients known to be different in a relevant way.

**Table 3:** Known-groups validity: median QOL-E scores by QOL-E Item 1 response.

QOL-E domain/summary score	Statistic	QOL-E Item 1 response			
		Excellent	Good	Acceptable	Poor
QOL-FIS	n	12	204	342	135
	Median (Q1, Q3)	88 (75, 100)	63 (50, 88)	50 (38, 63)	38 (13, 50)
QOL-FUN	n	13	203	338	133
	Median (Q1, Q3)	100 (89, 100)	89 (56, 100)	56 (22, 89)	22 (22, 33)
QOL-SOC	n	12	185	298	126
	Median (Q1, Q3)	100 (88, 100)	75 (50, 100)	50 (25, 75)	0 (0, 25)
QOL-SEX	n	11	183	281	104
	Median (Q1, Q3)	100 (83, 100)	83 (50, 100)	67 (42, 100)	50 (17, 100)
QOL-FAT	n	12	204	344	137
	Median (Q1, Q3)	95 (88, 100)	86 (81, 90)	76 (67, 86)	57 (52, 71)
QOL-MDSS	n	13	202	335	135
	Median (Q1, Q3)	93 (64, 93)	79 (67, 90)	57 (43, 74)	36 (21, 52)
QOL-GEN	n	11	183	292	120
	Median (Q1, Q3)	93 (65, 98)	78 (68, 87)	57 (42, 70)	35 (25, 47)
QOL-ALL	n	11	181	288	119
	Median (Q1, Q3)	92 (66, 96)	78 (71, 88)	57 (45, 69)	38 (24, 49)
QOL-TOI	n	12	199	327	130
	Median (Q1, Q3)	89 (71, 97)	77 (64, 87)	53 (40, 68)	34 (23, 44)

Q1, first quartile; Q3, third quartile; QOL-ALL, calculated by taking the mean of QOL-GEN and QOL-MDSS; QOL-FAT, fatigue; QOL-FIS, physical well-being; QOL-FUN, functional well-being; QOL-GEN, calculated by taking the mean of all domains except for QOL-MDSS; QOL-SEX, sexual well-being; QOL-SOC, social/family well-being; QOL-MDSS, MDS-specific disturbances; QOL-TOI, treatment outcome index calculated by taking the mean of QOL-FIS, QOL-FUN, and QOL-MDSS.

Item 1 of the QOL-E was “In general, you would say that your health is: excellent, good, acceptable, or poor”. Estimates are pooled across both baseline and Week 24. n is the number of observations (may include up to two observations from each patient, at baseline and Week 24).

#### Reliability

Test-retest reliability was performed on the subset of patients from the MEDALIST study. ICC values exceeded the prespecified acceptability threshold of 0.70 for all domains and summary scores except for QOL-FIS (0.66) and QOL-FUN (0.57) (**Table 4**). Internal consistency reliability, as assessed by Cronbach alpha and the omega coefficients, for the QOL-E domain scores and summary scores ranged from 0.69 to 0.80 for the alpha coefficient exceeding (or nearly exceeding) the prespecified acceptability threshold of 0.70; only QOL-FUN did not exceed the threshold (**Supplementary Table 6**). Removing item(s) from the QOL-FUN (Item 5), QOL-SOC (Item 7), and QOL-FAT domains (Items 11a or 12) led to a slight increase in the standardized alpha, indicating that these items may be redundant for the corresponding domains. Omega coefficients indicated findings similar to those seen in Cronbach alphas.

**Table 4:** Test-retest reliability: intraclass correlation coefficients.

QOL-E domain/summary score	n, ICC
QOL-FIS	121, 0.66
QOL-FUN	123, 0.57
QOL-SOC	109, 0.77
QOL-SEX	114, 0.81
QOL-FAT	123, 0.72
QOL-MDSS	121, 0.71
QOL-GEN	105, 0.84
QOL-ALL	104, 0.82
QOL-TOI	117, 0.77

ANOVA, analysis of variance; ICC, intraclass correlation coefficient; MDS, myelodysplastic neoplasms; QOL-ALL, calculated by taking the mean of QOL-GEN and QOL-MDSS; QOL-FAT, fatigue; QOL-FIS, physical well-being; QOL-FUN, functional well-being; QOL-GEN, calculated by taking the mean of all domains except for QOL-MDSS; QOL-SEX, sexual well-being; QOL-SOC, social/family well-being; QOL-MDSS, MDS-specific disturbances; QOL-TOI, treatment outcome index calculated by taking the mean of QOL-FIS, QOL-FUN, and QOL-MDSS.

The ICC is calculated using the two-way mixed-effect ANOVA model with interaction for the absolute agreement between single scores. Only patients with two distinct screening and baseline assessments in MEDALIST who reported the same response at both time points on Item 1 of the QOL-E were included.

Spearman correlations between all domains and summary scores of the QOL-E are shown in **Supplementary Table 7**. The directions of all correlations were generally consistent with the expectations: correlations were all moderate, with the exception of QOL-SEX, which tended to have much weaker correlations with other domains, indicating the homogeneity among these domains. Spearman correlations between the domains and corrected summary scores of the QOL-E (**Table 5**) were all moderate (0.3 ≤ r<0.7) or strong (r ≥0.7), ranging from 0.32 to 0.73, except for the correlation between QOL-SEX and corrected QOL-GEN (r=0.29). This suggests that the domains considered in each summary scale had good internal consistency, except for the QOL-SEX domain.

**Table 5:** Reliability: QOL-E domain and corrected summary score Spearman correlations.

QOL-E domain	QOL-GEN n r P value	QOL-ALL n r P value	QOL-TOI n r P value
QOL-FIS	609 0.58 <0.001	602 0.60 <0.001	671 0.56 <0.001
QOL-FUN	609 0.61 <0.001	602 0.63 <0.001	671 0.58 <0.001
QOL-SOC	609 0.65 <0.001	602 0.73 <0.001	-
QOL-SEX	515 0.29 <0.001	514 0.32 <0.001	-
QOL-FAT	609 0.73 <0.001	602 0.73 <0.001	-
QOL-MDSS	-	602 0.71 <0.001	671 0.60 <0.001

QOL-ALL, calculated by taking the mean of QOL-GEN and QOL-MDSS; QOL-FAT, fatigue; QOL-FIS, physical well-being; QOL-FUN, functional well-being; QOL-GEN, calculated by taking the mean of all domains except for QOL-MDSS; QOL-SEX, sexual well-being; QOL-SOC, social/family well-being; QOL-MDSS, MDS-specific disturbances; QOL-TOI, treatment outcome index calculated by taking the mean of QOL-FIS, QOL-FUN, and QOL-MDSS.

Correlations are pooled across both baseline and Week 24. Corrected summary score is calculated excluding the domain in question. n is the number of observations (may include up to two observations from each patient, at baseline and Week 24), r is the correlation coefficient, and P is the corresponding P value. Cells in dark gray indicate a weak correlation (<0.30), cells in medium gray indicate a moderate correlation (≥0.30 to<0.70), cells in blue gray indicate a strong correlation (≥0.70 to<0.90). No very strong correlations (≥0.90) were found.

#### Responsiveness (sensitivity to change)

Spearman correlation coefficients between the changes in the QOL-E domains/summary scores and changes in the potential anchors from baseline to Week 24 were all in expected directions (**Table 6**), except for that between the QOL-FAT domain and in RBC units transfused within previous 8 weeks; however, this correlation was near zero and not statistically significant (r=0.03, P=0.593). Correlations between changes in QOL-E domains/summary scores and changes in the patient-reported QOL-E Items 1 and 2 (“In general, you would say that your health is: excellent, good, acceptable, or poor” and “Compared to a month ago, your health is” [the absolute score was used as this item measure changes directly], respectively) and EORTC QLQ-C30 Items 29 and 30 (“How would you rate your overall health during the past week?” and “How would you rate your overall quality of life during the past week?”, respectively) mostly exceeded 0.3 (P<0.001) and were noticeably larger than correlations with changes in the clinical anchors (**Table 6**), with the exception of the QOL-SEX domain. This indicates that all QOL-E domains/summary scores, except for QOL-SEX, were able to detect changes perceived by the patients.

**Table 6:** Responsiveness: Spearman correlations between change in QOL-E domains/summary scores and changes in potential external anchors from Baseline to Week 24.

Domain	QOL-E Item 1 n r P value	QOL-E Item 2 n r P value	EORTC QLQ-C30 Item 29 n r P value	EORTC QLQ-C30 Item 30 n r P value	Hb level n r P value	RBC units transfused in the last 8 weeks n r P value
QOL-FIS	240 −0.36<0.001	241 −0.24 <0.001	217 0.35 <0.001	218 0.29 <0.001	195 0.26 <0.001	238 −0.04 0.496
QOL-FUN	238 −0.34 <0.001	240 −0.22 0.001	213 0.37 <0.001	214 0.31 <0.001	193 0.18 0.012	236 0.00 0.945
QOL-SOC	197 −0.34 <0.001	197 −0.17 0.014	176 0.30 <0.001	176 0.24 0.002	158 0.20 0.011	194 −0.02 0.738
QOL-SEX	196 −0.07 0.336	195 −0.05 0.511	186 0.17 0.021	187 0.18 0.012	153 0.00 0.952	195 −0.11 0.113
QOL-FAT	242 −0.40 <0.001	243 −0.38 <0.001	219 0.44 <0.001	220 0.42 <0.001	199 0.25 <0.001	242 0.03 0.593
QOL-MDSS	238 −0.37 <0.001	239 −0.28 <0.001	217 0.35 <0.001	218 0.32 <0.001	195 0.20 0.004	237 −0.16 0.015
QOL-GEN	192 −0.45 <0.001	193 −0.33 <0.001	173 0.47 <0.001	173 0.42 <0.001	153 0.27 0.001	189 −0.04 0.570
QOL-ALL	188 −0.46 <0.001	189 −0.38 <0.001	171 0.46 <0.001	171 0.43 <0.001	149 0.29 <0.001	185 −0.10 0.163
QOL-TOI	229 −0.45 <0.001	230 −0.32 <0.001	208 0.51 <0.001	209 0.43 <0.001	184 0.29 <0.001	226 −0.08 0.245

EORTC, European Organization for Research and Treatment of Cancer; Hb, hemoglobin; QLQ-C30, Quality of Life Questionnaire-Core 30; QoL, quality of life; QOL-ALL, calculated by taking the mean of QOL-GEN and QOL-MDSS; QOL-FAT, fatigue; QOL-FIS, physical well-being; QOL-FUN, functional well-being; QOL-GEN, calculated by taking the mean of all domains except for QOL-MDSS; QOL-SEX, sexual well-being; QOL-SOC, social/family well-being; QOL-MDSS, MDS-specific disturbances; QOL-TOI, treatment outcome index calculated by taking the mean of QOL-FIS, QOL-FUN, and QOL-MDSS; RBC, red blood cell.

Data from patients participating in the RevMDS study were excluded from the EORTC QLQ-C30 correlations as the EORTC QLQ-C30 was not administered in that study. n indicates the number of patients included in the analysis, r is the correlation coefficient, and P is the P value testing a non-zero correlation. Grey and white cells indicate correlation coefficients that were unacceptable (|r|<0.30) and acceptable (|r| ≥0.30), respectively.

Across all potential anchors, only the QOL-E Item 1 and EORTC QLQ-C30 Item 29 consistently yielded correlations greater than 0.3 across all the QOL-E domains, except for the QOL-SEX; therefore, they were chosen for the anchor-based analyses.

### Determination of MWPC thresholds

Plots of eCDF curves for the QOL-E domains/summary scores are shown in **Supplementary Figures 1–8**. All eCDF curves showed clear separations among the anchor groups for each domain/summary score, except for the QOL-FUN and QOL-SOC domains between the groups with ≥1 level of improvement and no change. These suggest estimates from the anchor groups with ≥1 level of improvement (deterioration) could be considered the triangulation of MWPC thresholds for most of the QOL-E domains/summary scores. For the QOL-SEX domain, MWPC thresholds were not triangulated as its responsiveness was not adequately demonstrated.

The distribution-based analysis was conducted on the subset of patients from the MEDALIST study. Per the triangulation approach specified, the range of MWPC thresholds for each QOL-E domain/summary score, as well as the proposed threshold for each direction (improvement/deterioration), are presented in **Table 7**.

**Table 7:** Triangulation of MWPC thresholds from anchor- and distribution-based analyses.

Statistics		QOL-FIS		QOL-FUN		QOL-SOC		QOL-FAT		QOL-MDSS		QOL-GEN		QOL-ALL		QOL-TOI	
		Imp.	Wors.	Imp.	Wors.	Imp.	Wors.	Imp.	Wors.	Imp.	Wors.	Imp.	Wors.	Imp.	Wors.	Imp.	Wors.
Imp./Wors. by ≥1 level on QOL-E Item 1	n	50	57	49	56	39	46	50	57	49	56	37	46	36	46	46	55
	Mean	14.8	−11.4	16.3	−21.6	13.5	−20.1	9.2	−8.7	15.0	−9.7	13.8	−12.2	14.8	−11.3	15.1	−14.8
	Median	12.5	−12.5	0.0	−22.2	0.0	−12.5	7.1	−4.8	11.9	−7.1	8.5	−11.3	10.0	−8.4	9.5	−13.0
Imp./Wors. by ≥1 level on QLQ-C30 Item 29	n	29	28	28	28	23	19	30	28	30	26	22	19	22	19	28	26
	Mean	15.1	−18.8	13.1	−24.6	8.7	−22.4	7.6	−18.5	11.6	−13.6	11.6	−18.5	11.2	−15.6	13.2	−19.5
	Median	12.5	−25.0	0.0	−16.7	0.0	0.0	7.1	−19.1	11.9	−15.5	7.1	−15.0	8.9	−15.1	7.1	−18.7
0.5×SD		10.7		16.2		18.8		7.0		11.9		10.5		10.6		10.3	
SEM		12.6		21.1		18.1		7.5		12.9		8.5		8.9		10.0	
Range of MWPC thresholds	(Imp.)	12.6 to 15.1		≥21.1		≥18.8		7.5 to 9.2		12.9 to 15.0		10.5 to 13.8		10.6 to 14.8		10.3 to 15.1	
	(Wors.)	−12.6 to −25.0		−21.6 to −24.6		−18.8 to −22.4		−7.5 to −19.1		−12.9 to 15.5		−11.3 to −18.5		−10.6 to −15.6		−13.0 to −19.5	
Minimum possible change		12.5		11.1		25.0		4.8		2.4		^a^		^a^		^a^	
Proposed MWPC threshold		≥12	≤−12	≥22	≤−22	≥25	≤−25	≥9	≤−9	≥14	≤−14	≥13	≤−13	≥13	≤−13	≥13	≤−13
Supporting statements		• Equivalent to the minimum possible change (rounded down) • ≥SEM • ≥0.5×SD		• Equivalent to twice the minimum possible change (rounded down) • ≥SEM • ≥0.5×SD		• Equivalent to the minimum possible change • ≥SEM • ≥0.5×SD		• Equivalent to twice the minimum possible change (rounded down) • ≥SEM • ≥0.5×SD		• Equivalent to a multiple (6) of the minimum possible change (rounded down) • ≥SEM • ≥0.5×SD		• A single threshold was chosen across the summary scores for practical purposes. • ≥SEM • ≥0.5×SD • Falls in the range of anchor-based estimates that are both ≥SEM and ≥0.5×SD					

EORTC, European Organization for Research and Treatment of Cancer; eCDF, empirical cumulative distribution function; Imp., improvement; MDS, myelodysplastic neoplasms; MWPC, meaningful within-patient change; QLQ-C30, Quality of Life Questionnaire-Core 30; QOL-ALL, calculated by taking the mean of QOL-GEN and QOL-MDSS; QOL-FAT, fatigue; QOL-FIS, physical well-being; QOL-FUN, functional well-being; QOL-GEN, calculated by taking the mean of all domains except for QOL-MDSS; QOL-SEX, sexual well-being; QOL-SOC, social/family well-being; QOL-MDSS, MDS-specific disturbances; QOL-TOI, treatment outcome index calculated by taking the mean of QOL-FIS, QOL-FUN, and QOL-MDSS; SD, standard deviation; SEM, standard error of measurement; Wors., worsening.

Data from patients participating in the RevMDS study were excluded from the EORTC QLQ-C30 anchor-based analyses as the EORTC QLQ-C30 was not administered in that study. Anchor-based estimates were not considered for improvement for the QOL-FUN and QOL-SOC (shaded in gray in table) as the eCDF curves from the no change and improvement by ≥1 level group did not show a clear separation.

a Minimum possible changes that an individual patient could experience were not considered in the triangulation of the QOL-E summary scores as the minima are too small (<0.1) to impact MWPC threshold estimates.

## Discussion

This analysis provides a psychometric evaluation of the QOL-E using data from four different clinical studies including more than 400 patients with Very low, Low, or Intermediate IPSS-R risk MDS or Low or Intermediate-1 IPSS risk MDS (20–25). In particular, the QOL-E was evaluated in terms of its distributional properties, convergent/divergent validity, known-group validity, reliability, and responsiveness (sensitivity to change). Anchor-based analyses were also performed to determine MWPC thresholds, with distribution-based analyses providing supportive evidence.

Results of this analysis indicated no problematic floor or ceiling effects for most QOL-E domains, with the exception of QOL-FUN (ceiling effect), QOL-SOC (ceiling effect), and QOL-SEX (both floor and ceiling effects). The convergent and divergent validity of all QOL-E domains and summary scores was adequately demonstrated. Most QOL-E domains/summary scores, however, showed weak correlations with clinical outcomes, such as Hb level, RBC units transfused, and platelet count. It should be noted that baseline Hb level has been shown to modify the impact of Hb improvements on PROs, resulting in correlations at or below 0.3 (43–46).

Additionally, as RBC transfusions were given on an as-needed basis while PROs were assessed at fixed-time intervals in the four studies included in this analysis, the impact of RBC transfusions on HRQoL and Hb may not have been consistently captured. The known-groups validity analysis revealed that most QOL-E domains could differentiate between subgroups of patients known to be different in an expected way.

The reliability of the QOL-E was also generally demonstrated by the results of the analysis. Test-retest reliability, which measures the consistency of scores over a short period of time, was demonstrated, with ICC values exceeding the acceptable threshold of 0.70 for all QOL-E domains/summary scores, except for QOL-FIS and QOL-FUN which fell slightly short. Additionally, internal consistency reliability estimates exceeded the prespecified acceptable threshold of 0.70 for all domains and summary scores except for QOL-FUN (0.69), suggesting that the items within each domain and domains within each summary scale are consistently measuring the same construct. Most QOL-E domains and summary scores, excluding the QOL-SEX domain, showed an adequate ability to detect changes in the selected anchors, making it a reliable tool for tracking changes in quality of life over time. The suboptimal psychometric performance of QOL-SEX domain was not unexpected, which is why the summary scores of QOL-E (QOL-GEN and QOL-ALL) can be calculated without QOL-SEX (see “Scoring of the QOL-E” in the **Supplementary Material**). Nevertheless, QOL-SEX has been retained in the QOL-E to capture this dimension of patients’ experiences and perspectives in clinical practice.

All eCDF curves in this analysis showed clear and consistent separations among the anchor groups for each domain/summary score with the exception of the QOL-FUN and QOL-SOC domains for groups with ≥1 level of improvement or no change. Ultimately, thresholds for improvement and worsening, respectively, are proposed for the QOL-FIS (≥12 and ≤−12), QOL-FUN (≥22 and ≤−22), QOL-SOC (≥25 and ≤−25), QOL-FAT (≥9 and ≤−9), and QOL-MDSS (≥14 and ≤−14), with thresholds of ≥13 and ≤−13 proposed for the QOL-GEN, QOL-ALL, and QOL-TOI.

Certain limitations of this analysis should be noted. First, two different versions of the QOL-E (versions 2 and 3) were used in the various studies included in the analysis; however, the differences included only minor wording changes, as noted in the Methods, *Study design and outcome assessment* section. In addition to test-retest reliability and distribution-based analysis, all other analyses (convergent and divergent validity, known-groups validity, reliability, responsiveness, and triangulation of MWPC thresholds) were also conducted in the subgroup of patients from the MEDALIST study (i.e., using version 3 of the QOL-E) and results and conclusions (data not shown) were consistent with those presented here. Second, there are slight differences in the recall period used in the QOL-E questions and those of the anchors. In particular, the QOL-E Items 1 (used as an anchor), 6, 7, and 14 ask about current or general conditions without specifying a recall period, while Items 3, 4, 5, 8, 9, 10, 11, 12, and 13 ask patients about their experiences over the past week. The EORTC QLQ-C30 Item 29 anchor also asks patients about their health over the past week. The impact that this may have on the findings of this analysis is not completely certain, but it is likely to be minor as a 1-week recall period is relatively short. Third, this analysis included 20 patients who had a High IPSS-R score and 1 patient who had a Very-high IPSS-R score. The presence of patients with IPSS-R scores of High and Very high is a consequence of converting IPSS scores to IPSS-R scores for the DARB-MDS, RevMDS and EQoL-MDS studies. However, given this is a small sample (4.6%) of patients, it is unclear whether the analysis findings may be generalizable to patients with higher-risk MDS. Moreover, the majority of patients (84.0%) included in this analysis were White. Ensuring equitable inclusion of different racial and ethnic groups in the study sample is essential for minimizing disparities; nevertheless, this does not imply that the instrument cannot be used to assess QoL in diverse populations. Finally, as data from four different protocols were used, data collection (including timing) and standardization likely differed. Although data were aligned as much as possible, differences between studies still exist and this may have introduced additional variation into the analyses. To evaluate the impact this may have had on the results, as mentioned above, all analyses were also conducted on the MEDALIST population alone and results did not alter any conclusions.

The study evaluated the psychometric performance of the QOL-E in assessing HRQoL in patients with LR-MDS and determined the thresholds for defining MWPC (improvement and worsening) in QOL-E domain and summary scores. Overall, the QOL-E showed acceptable psychometric properties across most domains/summary scores with the exception of the QOL-SEX domain, which did not meet the necessary criteria for known-groups validity and responsiveness. MWPC thresholds for improvement and worsening for all other QOL-E domains and the three summary scores are proposed. The study results demonstrate that the QOL-E is generally fit for purpose to assess treatment effects in populations with LR-MDS and the proposed MWPC thresholds can be used to assess within-patient treatment effect on HRQoL, as assessed by the QOL-E, in future studies.

## Data Availability

The original contributions presented in the study are included in the article/**Supplementary Material**. Further inquires can be directed to Bristol Myers Squibb; the policy on data sharing may be found at https://www.bms.com/researchers-and-partners/clinical-trials-and-research/disclosure-commitment.html.
