# Supplementary material for: Psychometric properties and meaningful change thresholds for the QOL-E instrument in patients with myelodysplastic neoplasms
Source: Front Oncol. 2025 Feb 7;15:1507854. doi: 10.3389/fonc.2025.1507854 (PMC11842348; doi:10.3389/fonc.2025.1507854)
Supplement: Supplementary file 1 [file DataSheet1.docx]

# Supplementary Material

## Scoring of the QOL-E

To score each domain and composite summary score, the following steps were performed:

1. Some items of the QOL-E questionnaire (i.e., Items 1–2, 4, and 6–14) were first rescaled so that better health corresponded with a higher numerical value across all items. For example, Item 1=5 was a raw response score (RS) for Item 1. For Items 6a–c (which have three response levels per item: true, false, and I don’t know), a response of “I don’t know” was considered a “missing” response.
2. Each item was transformed into a standardized 0-to-100 scale according to the following formula: Standardized Item Score=[(actual RS [or rescaled score per Step 1 above]–lowest possible RS) / possible RS range]×100.
3. The score for each domain was calculated if responses were given to more than 50% of the items in that domain; otherwise, it was set to missing. For example, if a patient answered only two of four questions for the physical domain, they would have a missing score for that given assessment visit. If a patient answered four of seven questions for fatigue, they would have a score for the fatigue domain by averaging across all the non-missing standardized item scores within the fatigue domain [i.e., fatigue (QOL-FAT)=(Item 9+Item 10+Item 11a+Item 11b+Item 11c+Item 11d+Item 12) / 7; assuming no missing item scores]. No item was weighted more heavily than another.
4. Scores for a specific composite summary score (i.e., QOL-GEN, QOL-ALL, or QOL-TOI) were calculated as follows:
   1. QOL-GEN=(physical well-being [QOL-FIS]+functional well-being [QOL-FUN]+social/family well-being [QOL-SOC]+sexual well-being [QOL-SEX]+QOL-FAT) / 5. If any domain score was missing, the QOL-GEN was set to missing, except when only QOL-SEX was missing, in which case the score was calculated as: (QOL-FIS+QOL FUN+QOL-SOC+QOL-FAT)/4.
   2. QOL-ALL=(QOL-GEN+MDS-specific disturbances [QOL-MDSS]) / 2. If any domain score was missing, the QOL-ALL was set to missing, except when only QOL-SEX was missing, in which case the score was calculated using the QOL-GEN without QOL-SEX as calculated above.
   3. QOL-TOI=(QOL-FIS+QOL-FUN+QOL-MDSS) / 3. If any domain score was missing, the QOL-TOI was set to missing.

## SUPPLEMENTARY TABLE 1 Summary of study characteristics.

| **Study** | **Key inclusion criteria** | **Treatment(s)** | **Sample size^a^** | **PRO instrument** | **PRO assessment schedule** |
| --- | --- | --- | --- | --- | --- |
| **MEDALIST (1, 2)** | 1) Age ≥18 years  2) MDS IPSS-R Very low-/Low-/Intermediate-risk  3) Intolerant to/ineligible for ESA  4) RBC TD with average TB ≥2 units/8 wks for ≥16 wks before randomization  5) Hb ≤10.0 g/dL  6) No consecutive 56-day period TF during 16 wks prior to randomization | Luspatercept 1.0 mg/kg SC on Day 1 of each 21-day cycle vs. placebo | N=229: luspatercept=153 and placebo=76 | EORTC-QLQ-C30  QOL-E (version 3) | Screening, baseline, and every 6 weeks |
| **DARB-MDS (3)** | 1) MDS IPSS Low/Intermediate-1 risk  2) Hb <11.0 g/dL and symptoms of anemia  3) No evidence of other causes of anemia, and adequate liver and kidney function | DARB SC injections 150 μg/wk (up to 300 μg/wk) for up to 24 wks | N=41:  TF=24 and  TD=17 | EORTC-QLQ-C30  QOL-E (version 2) | Baseline, Weeks 4, 8, 18, and 24 |
| **EQoL-MDS (4)** | 1) Age ≥18 years  2) MDS IPSS Low/Intermediate-1 risk  3) Platelet count <30×10⁹/L  4) Refractory, ineligible for alternative medications, or relapse while receiving them | Eltrombopag (50 mg daily up to 300 mg daily) or placebo up to 24 wks until progression | N=169:  eltrombopag=112 and  Placebo=57 | EORTC-QLQ-C30  QOL-E (version 3) | Baseline and every 3 months |
| **QoL-ESC RevMDS (5)** | 1) Age ≥18 years  2) Primary MDS IPSS Low/Intermediate-1 risk  3) Hb <10 g/dL  4) del(5q) abnormality  5) No evidence of other causes of anemia | Lenalidomide 10 mg daily for up to 52 wks | N=45 | QOL-E (version 2) only | Baseline, Weeks 8, 12, and 24; Week 52 (second phase only) |

DARB, darbepoetin; EORTC, European Organization for Research and Treatment of Cancer; ESA, erythropoiesis-stimulating agent; Hb, hemoglobin; IPSS, International Prognostic Scoring System; IPSS-R, Revised International Prognostic Scoring System; MDS, myelodysplastic neoplasms; PRO, patient-reported outcome; QLQ-C30, Quality of Life Questionnaire-Core 30; RBC, red blood cell; SC, subcutaneous; TB, transfusion burden; TD, transfusion-dependent; TF, transfusion-free.

^a^Includes all patients reported in the respective publication.

## SUPPLEMENTARY TABLE 2 Recall periods for each item included in the six QOL-E domains.

| **Domain** | **Item** | **Recall period** |
| --- | --- | --- |
| **QOL-FIS** | Items 3a–d | 1 week |
| **QOL-FUN** | Items 4a–b  Item 5 | 1 week |
| **QOL-SOC** | Items 6a–c and Item 7 | Not specified^a^ |
| **QOL-SEX** | Item 8  Item 14f | 1 week  Not specified^a^ |
| **QOL-FAT** | Item 9  Item 10  Items 11a–d  Item 12 | 1 week |
| **QOL-MDSS** | Item 13  Items 14a–e  Item 14g | 1 week  Not specified^a^  Not specified^a^ |

QOL-FAT, fatigue; QOL-FIS, physical well-being; QOL-FUN, functional well-being, QOL-SEX, sexual well-being; QOL-SOC, social/family well-being; QOL-MDSS, MDS-specific disturbances.

^a^The item asks about the patient’s present state.

## SUPPLEMENTARY TABLE 3 Response categorizations by anchor for responsiveness and anchor-based analyses.

| **Anchor^a^** | **Improvement by ≥2 levels** | **Improvement by 1 level** | **No change** | **Worsening by 1 level** | **Worsening by ≥2 levels** | **Source** |
| --- | --- | --- | --- | --- | --- | --- |
| **QOL-E Item 1** | =−2 or −3 | =−1 | =0 | =1 | =2 or 3 |  |
| **QOL-E Item 2 (absolute score)^b^** | N/A | =1 (improved) | =2 (the same) | =3 (worse) | =4 (much worse) |  |
| **EORTC QLQ-C30 Item 29** | ≥3 | =2 | ≤1 to ≥−1 | =−2 | ≤−3 | Bedard, 2014 (6) |
| **EORTC QLQ-C30 Item 30^b^** | ≥3 | = | ≤1 to ≥−1 | =−2 | ≤−3 | Bedard, 2014 (6) |
| **Hb level (1)^b^** | ≥2 g/dL | ≥1 to <2 g/dL | >−1 to <1 g/dL | >−2 to ≤−1 g/dL | ≤−2 g/dL |  |
| **Hb level (2)^b^** | N/A | ≥ 1.5 g/dL | > −1.5 to < 1.5 g/dL | ≤ −1.5 g/dL | N/A |  |
| **RBC transfusion burden level (1)^b^** | ≤−2 RBC units/8 wks | >−2 to ≤−1 RBC units/8 wks | >−1 to <1 RBC units/8 wks | ≥1 to <2 RBC units/8 wks | ≥2 RBC units/8 wks | Platzbecker, 2019 (7) |
| **RBC transfusion burden level (2)^b^** | N/A | ≤−2 RBC units/8 wks | >−2 to <2 RBC units/8 wks | ≥2 RBC units/8 wks | N/A | Platzbecker, 2019 (7) |

EORTC, European Organization for the Research and Treatment of Cancer, Hb, hemoglobin, N/A, not applicable, QLQ-C30, Quality of Life Questionnaire – Core 30; RBC, red blood cell.

^a^The measures included the QOL-E Item 1 (“In general, you would say that your health is”), QOL-E Item 2 (“Compared to a month ago, your health is”; the absolute score was used as this item measure changes directly), EORTC QLQ-C30 Item 29 (“How would you rate your overall health during the past week?”), EORTC QLQ-C30 Item 30 (“How would you rate your overall quality of life during the past week?”), Hb level, and RBC transfusion burden level.

^b^Indicates potential anchors were prespecified, but not included in anchor-based threshold analyses as the absolute value of the correlation with the QOL-E did not exceed the prespecified threshold of 0.3.

## SUPPLEMENTARY TABLE 4 Distribution of QOL-E scores at baseline.

| **Domain/statistic** | **Analysis population (N=458)** |
| --- | --- |
| **QOL-FIS** | |
| n | 448 |
| Mean (SD) | 52.5 (24.4) |
| Median | 50.0 |
| Q1, Q3 | 37.5, 62.5 |
| Min, Max | 0, 100 |
| Floor, n (%)^a^ | 18 (4.0%) |
| Ceiling, n (%)^a^ | 22 (4.9%) |
| Missing, n (%) | 10 (2.2%) |
| **QOL-FUN** | |
| n | 444 |
| Mean (SD) | 55.8 (33.8) |
| Median | 55.6 |
| Q1, Q3 | 22.2, 94.4 |
| Min, Max | 0, 100 |
| Floor, n (%)^a^ | 8 (1.8%) |
| Ceiling, n (%)^a^ | 111 (25.0%) |
| Missing, n (%) | 14 (3.1%) |
| **QOL-SOC** | |
| n | 402 |
| Mean (SD) | 47.5 (37.1) |
| Median | 50.0 |
| Q1, Q3 | 0, 75.0 |
| Min, Max | 0, 100 |
| Floor, n (%)^a^ | 101 (25.1%) |
| Ceiling, n (%)^a^ | 82 (20.4%) |
| Missing, n (%) | 56 (12.2%) |
| **QOL-SEX** | |
| n | 369 |
| Mean (SD) | 64.0 (35.3) |
| Median | 66.7 |
| Q1, Q3 | 41.7, 100 |
| Min, Max | 0, 100 |
| Floor, n (%)^a^ | 45 (12.2%) |
| Ceiling, n (%)^a^ | 138 (37.4%) |
| Missing, n (%) | 89 (19.4%) |
| **QOL-FAT** | |
| n | 455 |
| Mean (SD) | 74.0 (16.3) |
| Median | 76.2 |
| Q1, Q3 | 61.9, 85.7 |
| Min, Max | 14.3, 100 |
| Floor, n (%)^a^ | 0 (0%) |
| Ceiling, n (%)^a^ | 19 (4.2%) |
| Missing, n (%) | 3 (0.7%) |
| **QOL-MDSS** | |
| n | 444 |
| Mean (SD) | 58.5 (24.4) |
| Median | 59.6 |
| Q1, Q3 | 40.5, 78.6 |
| Min, Max | 0, 100 |
| Floor, n (%)^a^ | 1 (0.2%) |
| Ceiling, n (%)^a^ | 13 (2.9%) |
| Missing, n (%) | 14 (3.1%) |
| **QOL-GEN** | |
| n | 388 |
| Mean (SD) | 58.5 (22.2) |
| Median | 58.7 |
| Q1, Q3 | 41.2, 77.4 |
| Min, Max | 8.3, 100 |
| Floor, n (%)^a^ | 0 (0%) |
| Ceiling, n (%)^a^ | 2 (0.5%) |
| Missing, n (%) | 70 (15.3%) |
| **QOL-ALL** | |
| n | 384 |
| Mean (SD) | 58.7 (21.6) |
| Median | 59.4 |
| Q1, Q3 | 43.2, 75.8 |
| Min, Max | 12.5, 99.5 |
| Floor, n (%)^a^ | 0 (0%) |
| Ceiling, n (%)^a^ | 0 (0%) |
| Missing, n (%) | 74 (16.2%) |
| **QOL-TOI** | |
| n | 427 |
| Mean (SD) | 55.8 (22.1) |
| Median | 56.0 |
| Q1, Q3 | 39.2, 74.0 |
| Min, Max | 4.8, 100 |
| Floor, n (%)^a^ | 0 (0%) |
| Ceiling, n (%)^a^ | 2 (0.5%) |
| Missing, n (%) | 31 (6.8%) |

Max, maximum; MDS, myelodysplastic neoplasms; Min, minimum; Q1, first quartile; Q3, third quartile, QOL-ALL, calculated by taking the mean of QOL-GEN and QOL-MDSS; QOL-FAT, fatigue; QOL-FIS, physical well-being; QOL-FUN, functional well-being, QOL-GEN, calculated by taking the mean of all domains except for QOL-MDSS; QOL-SEX, sexual well-being; QOL-SOC, social/family well-being; QOL-MDSS, MDS-specific disturbances; QOL-TOI, treatment outcome index calculated by taking the mean of QOL-FIS, QOL-FUN, and QOL-MDSS; SD, standard deviation.

^a^Denominators for floor and ceiling percentages are the numbers of patients with non-missing scores, while the denominator of the missing percentages is the number of patients in the analysis population (n=458).

4

## SUPPLEMENTARY TABLE 5 Known-groups validity: mean QOL-E scores by transfusion dependency group.

| **QOL-E domain/summary score** | **Statistic** | **RBC transfusion dependency** | |
| --- | --- | --- | --- |
|  |  | **Dependent** | **Independent** |
| **QOL-FIS** | n | 447 | 236 |
|  | Median (Q1, Q3) | 50.0 (37.5, 62.5) | 62.5 (50.0, 75.0) |
| **QOL-FUN** | n | 445 | 233 |
|  | Median (Q1, Q3) | 55.6 (22.2, 88.9) | 66.7 (22.2, 100) |
| **QOL-SOC** | n | 405 | 206 |
|  | Median (Q1, Q3) | 50.0 (0.0, 75.0) | 50.0 (25.0, 100) |
| **QOL-SEX** | n | 391 | 184 |
|  | Median (Q1, Q3) | 66.7 (41.7, 100) | 75.0 (41.7, 100) |
| **QOL-FAT** | n | 451 | 238 |
|  | Median (Q1, Q3) | 76.2 (61.9, 85.7) | 81.0 (66.7, 90.5) |
| **QOL-MDSS** | n | 447 | 230 |
|  | Median (Q1, Q3) | 57.1 (35.7, 73.8) | 66.7 (50.0, 85.7) |
| **QOL-GEN** | n | 400 | 197 |
|  | Median (Q1, Q3) | 56.3 (40.6, 74.6) | 67.0 (48.7, 83.1) |
| **QOL-ALL** | n | 398 | 192 |
|  | Median (Q1, Q3) | 55.8 (41.0, 74.2) | 68.7 (51.9, 82.9) |
| **QOL-TOI** | n | 439 | 219 |
|  | Median (Q1, Q3) | 51.3 (37.8, 70.8) | 65.7 (44.9, 83.5) |

MDS, myelodysplastic neoplasms ; Q1, first quartile; Q3, third quartile; QOL-ALL, calculated by taking the mean of QOL-GEN and QOL-MDSS; QOL-FAT, fatigue; QOL-FIS, physical well-being; QOL-FUN, functional well-being, QOL-GEN, calculated by taking the mean of all domains except for QOL-MDSS; QOL-SEX, sexual well-being; QOL-SOC, social/family well-being; QOL-MDSS, MDS-specific disturbances; QOL-TOI, treatment outcome index calculated by taking the mean of QOL-FIS, QOL-FUN, and QOL-MDSS; RBC, red blood cell.

Estimates are pooled across both baseline and Week 24. n is the number of observations (may include up to two observations from each patient, at baseline and Week 24).

## SUPPLEMENTARY TABLE 6 Internal consistency: Cronbach alpha and the omega coefficient.

| **QOL-E domain/summary score or Item** | **Correlation with total of remaining items** | **Cronbach alpha** | **Omega** |
| --- | --- | --- | --- |
| **QOL-FIS** | - | 0.74 | 0.76 |
| Deleted Item | | | |
| Item 3a | 0.45 | 0.73 | - |
| Item 3b | 0.63 | 0.63 | - |
| Item 3c | 0.62 | 0.64 | - |
| Item 3d | 0.45 | 0.73 | - |
| **QOL-FUN** | - | 0.69 | 0.65 |
| Deleted Item | | | |
| Item 4a | 0.58 | 0.48 | - |
| Item 4b | 0.56 | 0.52 | - |
| Item 5^a^ | 0.37 | 0.75 | - |
| **QOL-SOC** | - | 0.77 | 0.78 |
| Deleted Item | | | |
| Item 6a | 0.65 | 0.68 | - |
| Item 6b | 0.69 | 0.66 | - |
| Item 6c | 0.55 | 0.73 | - |
| Item 7^a^ | 0.43 | 0.79 | - |
| **QOL-SEX** | - | 0.72 | 0.71 |
| **QOL-FAT** | - | 0.76 | 0.79 |
| Deleted Item | | | |
| Item 9 | 0.67 | 0.69 | - |
| Item 10 | 0.68 | 0.68 | - |
| Item 11a^a^ | 0.25 | 0.78 | - |
| Item 11b | 0.38 | 0.75 | - |
| Item 11c | 0.60 | 0.70 | - |
| Item 11d | 0.52 | 0.72 | - |
| Item 12^a^ | 0.28 | 0.77 | - |
| **QOL-MDSS** | - | 0.80 | 0.80 |
| Deleted Item | | | |
| Item 13 | 0.44 | 0.79 | - |
| Item 14a | 0.54 | 0.77 | - |
| Item 14b | 0.56 | 0.77 | - |
| Item 14c | 0.58 | 0.76 | - |
| Item 14d | 0.59 | 0.76 | - |
| Item 14e | 0.59 | 0.76 | - |
| Item 14g | 0.42 | 0.79 | - |
| **QOL-GEN** | - | - | 0.77 |
| **QOL-ALL** | - | - | 0.83 |
| **QOL-TOI** | - | - | 0.75 |

MDS, myelodysplastic neoplasms; QOL-ALL, calculated by taking the mean of QOL-GEN and QOL-MDSS; QOL-FAT, fatigue; QOL-FIS, physical well-being; QOL-FUN, functional well-being, QOL-GEN, calculated by taking the mean of all domains except for QOL-MDSS; QOL-SEX, sexual well-being; QOL-SOC, social/family well-being; QOL-MDSS, MDS-specific disturbances; QOL-TOI, treatment outcome index calculated by taking the mean of QOL-FIS, QOL-FUN, and QOL-MDSS.

^a^Removing item(s) from the QOL-FUN (Item 5), QOL-SOC (Item 7), and QOL-FAT domains (Items 11a or 12) led to a slight increase in the standardized alphas; however, the increases were small in magnitude potentially indicating that the items may be redundant, but not conclusively.

## SUPPLEMENTARY TABLE 7 Reliability: QOL-E inter-domain correlations.

| **QOL-E Domain** | **QOL-FIS n, r, P** | **QOL-FUN n, r, P** | **QOL-SOC n, r, P** | **QOL-SEX n, r, P** | **QOL-FAT n, r, P** | **QOL-MDSS n, r, P** |
| --- | --- | --- | --- | --- | --- | --- |
| **QOL-FIS** | - | 686, 0.47, <0.001 | 618, 0.52, <0.001 | 577, 0.17, <0.001 | 694, 0.58, <0.001 | 681, 0.50, <0.001 |
| **QOL-FUN** | 686, 0.47, <0.001 | - | 614, 0.56, <0.001 | 572, 0.21, <0.001 | 688, 0.67, <0.001 | 677, 0.54, <0.001 |
| **QOL-SOC** | 618, 0.52, <0.001 | 614, 0.56, <0.001 | - | 525, 0.30, <0.001 | 623, 0.58, <0.001 | 615, 0.66, <0.001 |
| **QOL-SEX** | 577, 0.17, <0.001 | 572, 0.21, <0.001 | 525, 0.30, <0.001 | - | 581, 0.25, <0.001 | 580, 0.29, <0.001 |
| **QOL-FAT** | 694, 0.58, <0.001 | 688, 0.67, <0.001 | 623, 0.58, <0.001 | 581, 0.25, <0.001 | - | 688, 0.60, <0.001 |
| **QOL-MDSS** | 681, 0.50, <0.001 | 677, 0.54, <0.001 | 615, 0.66, <.0001 | 580, 0.29, <0.001 | 688, 0.60, <0.001 | - |

MDS, myelodysplastic neoplasms; QOL-FAT, fatigue; QOL-FIS, physical well-being; QOL-FUN, functional well-being, QOL-SEX, sexual well-being; QOL-SOC, social/family well-being; QOL-MDSS, MDS-specific disturbances.

Estimates are pooled across both baseline and Week 24. n is the number of observations (may include up to two observations from each patient, at baseline and Week 24), r is the correlation coefficient, and P is the corresponding P value. Cells in dark gray indicate a weak correlation (<0.30) and cells in medium gray indicate a moderate correlation (≥0.30 to <0.70). No strong (≥0.70 to <0.90) or very strong (≥0.90) correlations were found.

## SUPPLEMENTARY FIGURE 1 eCDF of change from baseline to Week 24 on QOL-FIS.


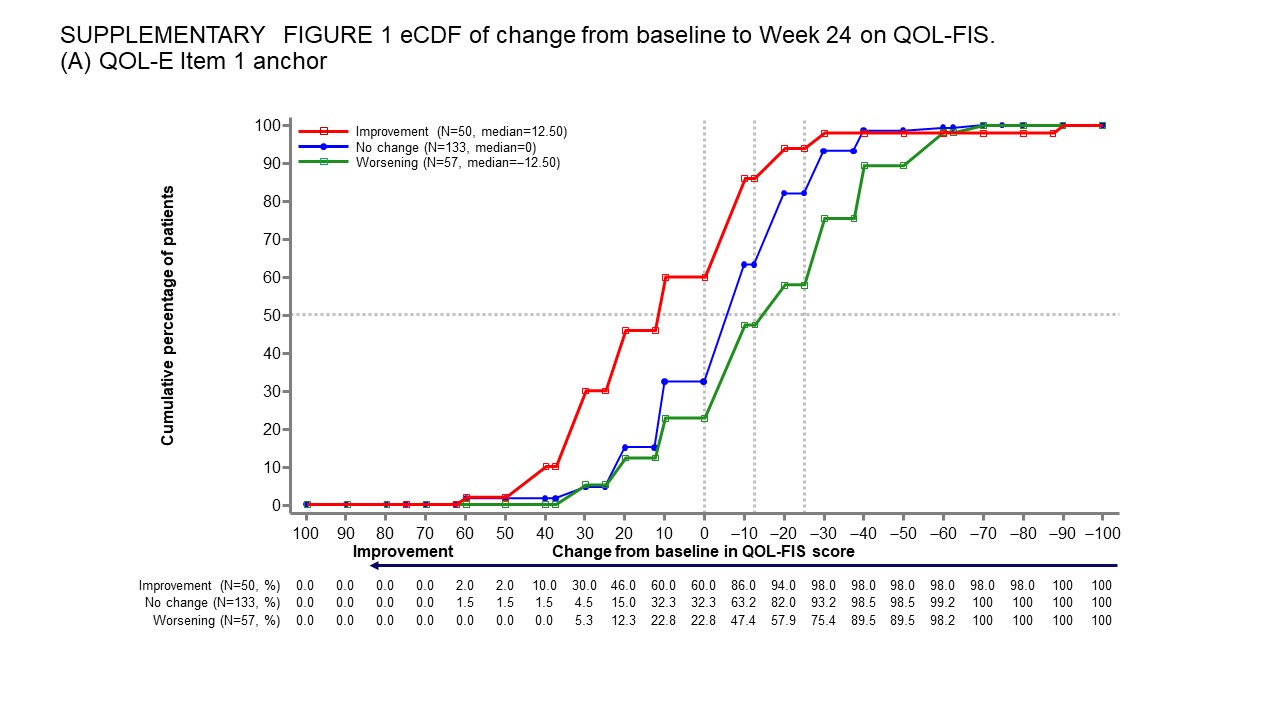


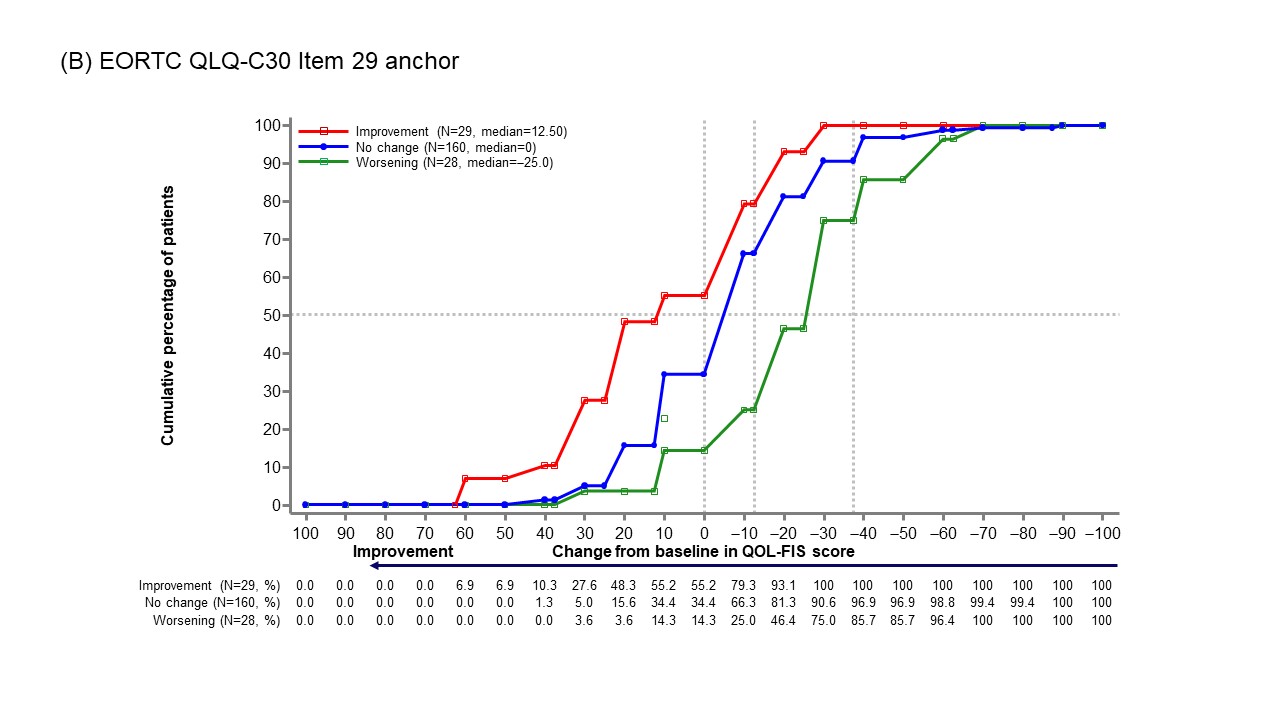


eCDF, empirical cumulative distribution function; EORTC, European Organization for Research and Treatment of Cancer; QLQ-C30, Quality of Life Questionnaire-Core 30; QOL-FIS, physical well-being.

Data from patients participating in the RevMDS study were excluded from the EORTC QLQ-C30 anchor-based analyses as the EORTC QLQ-C30 was not administered in that study.

## SUPPLEMENTARY FIGURE 2 eCDF of change from baseline to Week 24 on QOL-FUN.


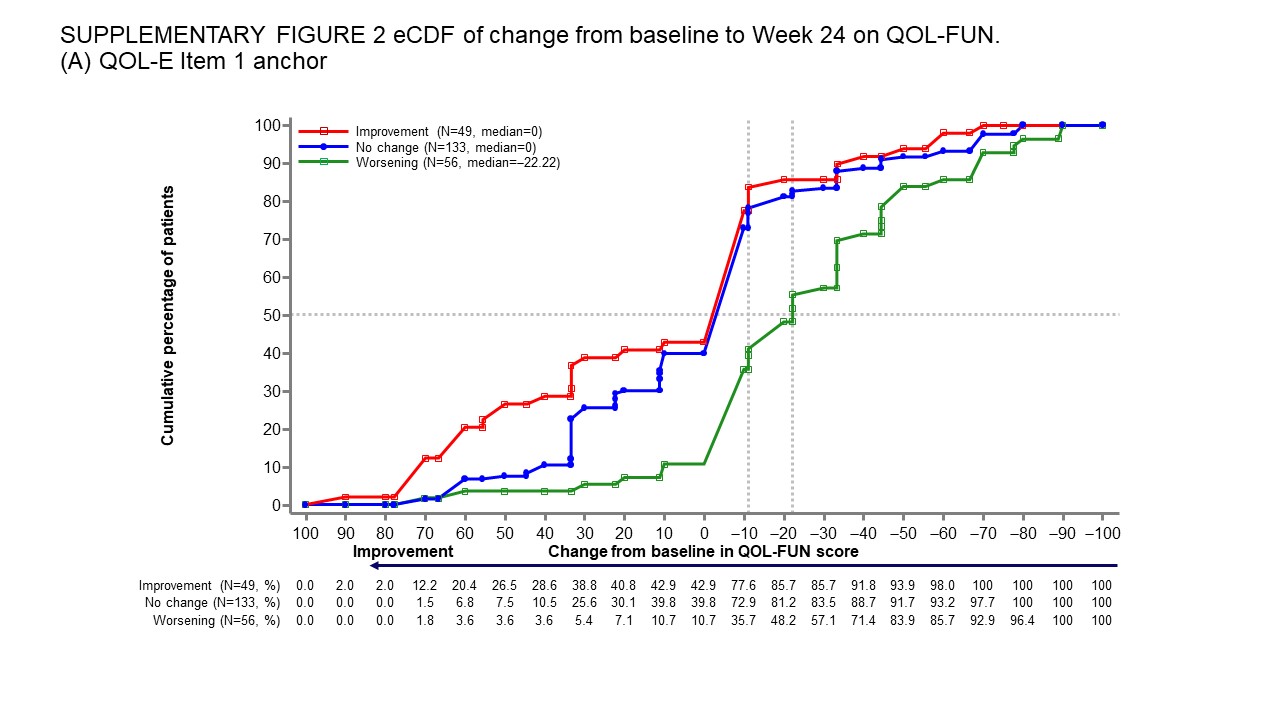


**
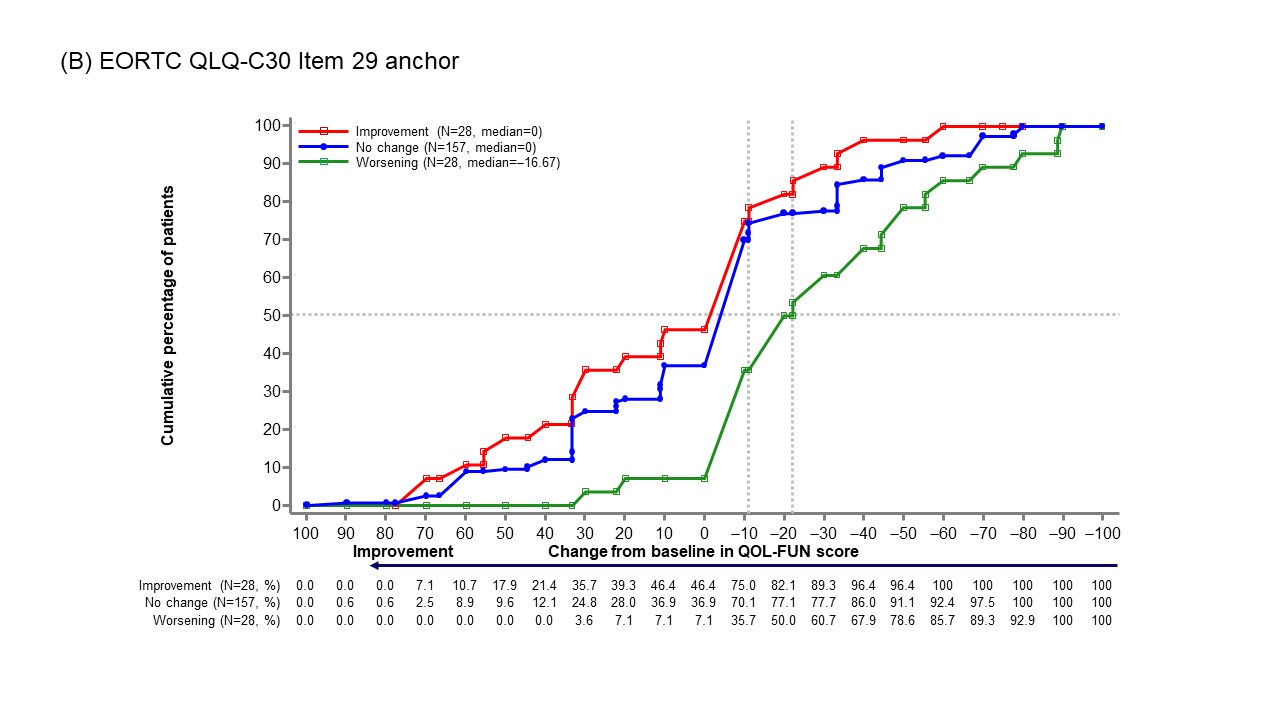
**

eCDF, empirical cumulative distribution function; EORTC, European Organization for Research and Treatment of Cancer; QLQ-C30, Quality of Life Questionnaire-Core 30; QOL-FUN, functional well-being.

Data from patients participating in the RevMDS study were excluded from the EORTC QLQ-C30 anchor-based analyses as the EORTC QLQ-C30 was not administered in that study.

SUPPLEMENTARY FIGURE 3 eCDF of change from baseline to Week 24 on QOL-SOC.


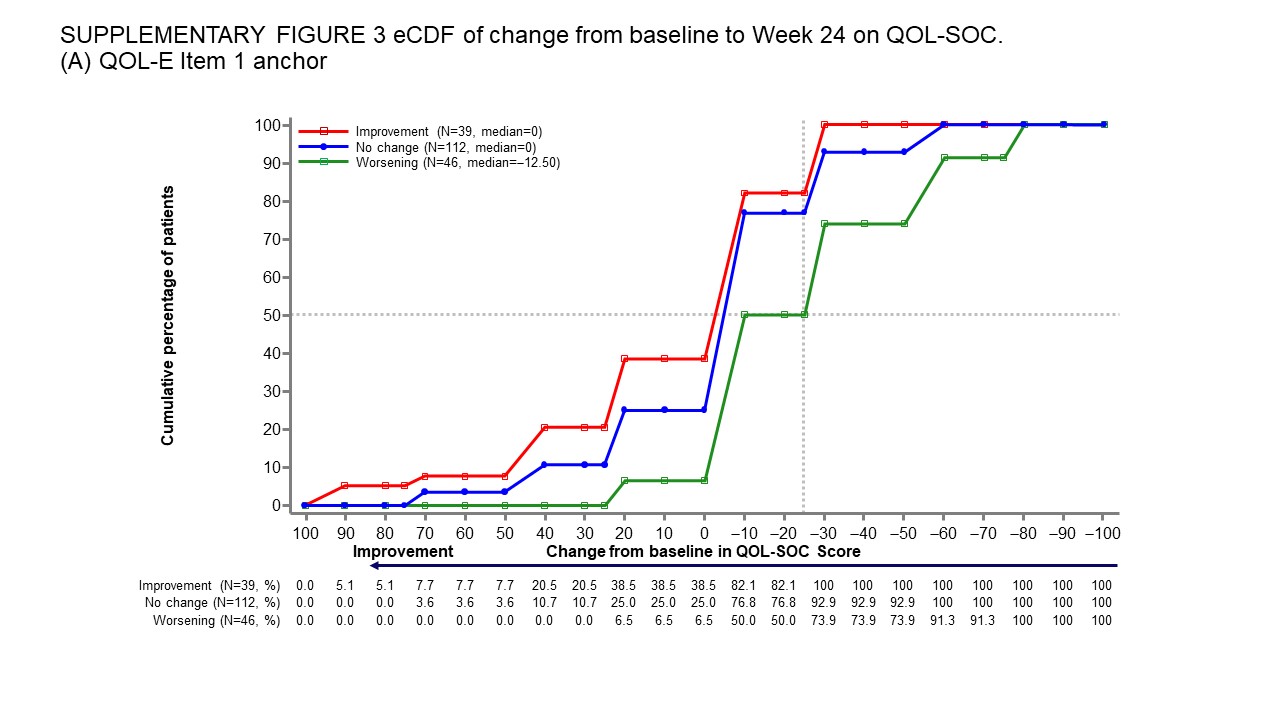


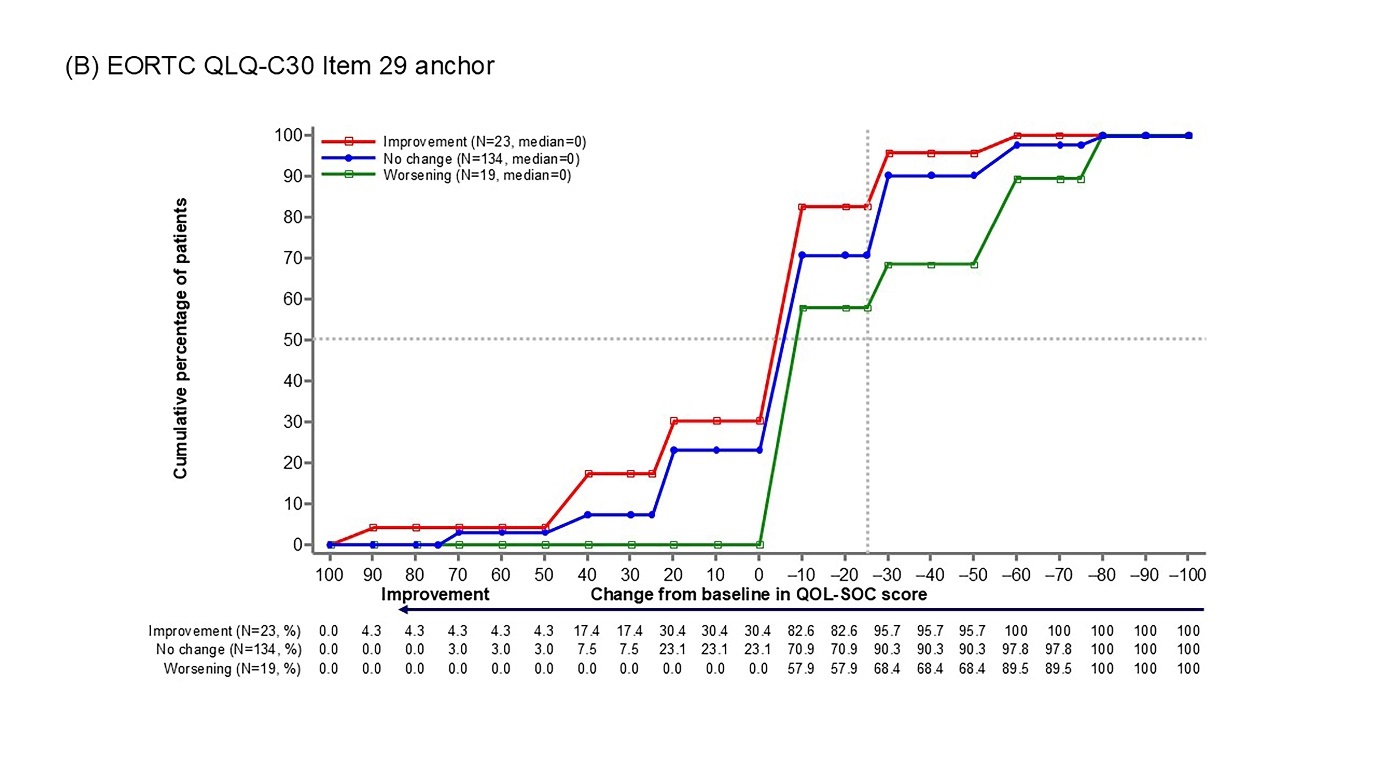


eCDF, empirical cumulative distribution function; EORTC, European Organization for Research and Treatment of Cancer; QLQ-C30, Quality of Life Questionnaire-Core 30; QOL-SOC, social/family well-being.

Data from patients participating in the RevMDS study were excluded from the EORTC QLQ-C30 anchor-based analyses as the EORTC QLQ-C30 was not administered in that study.

## SUPPLEMENTARY FIGURE 4 eCDF of change from baseline to Week 24 on QOL-FAT.

**
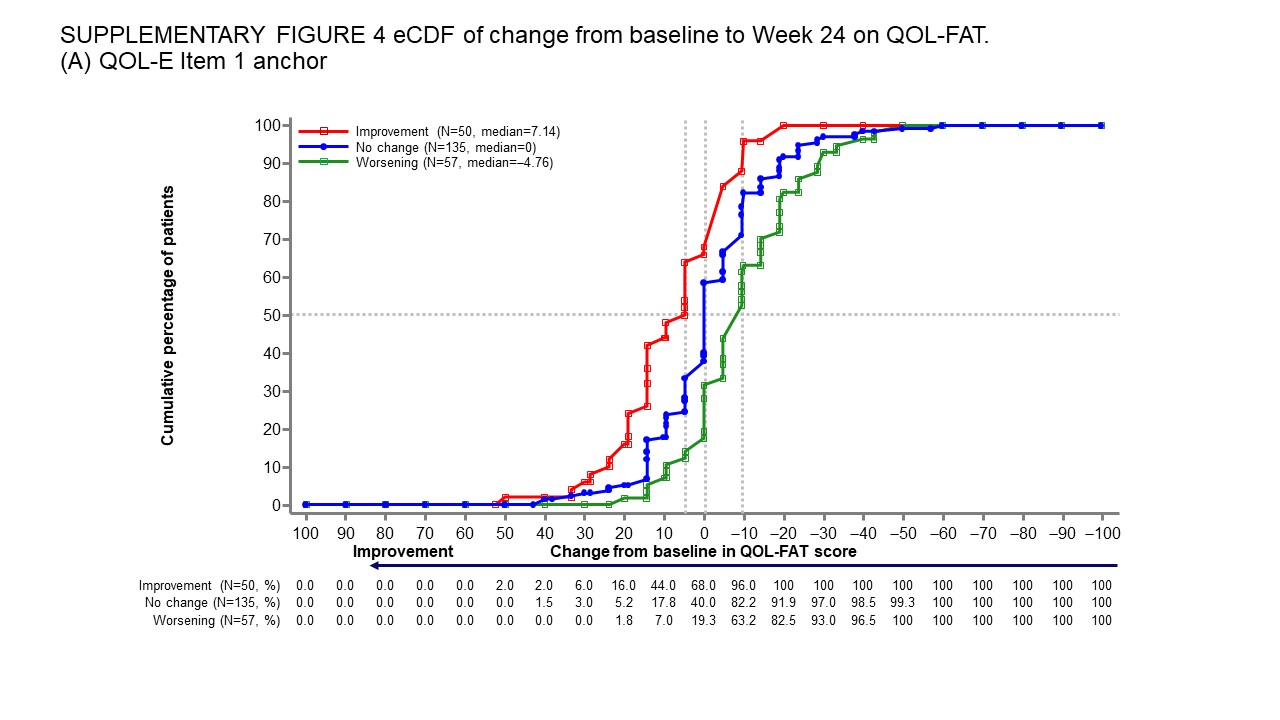
**

**
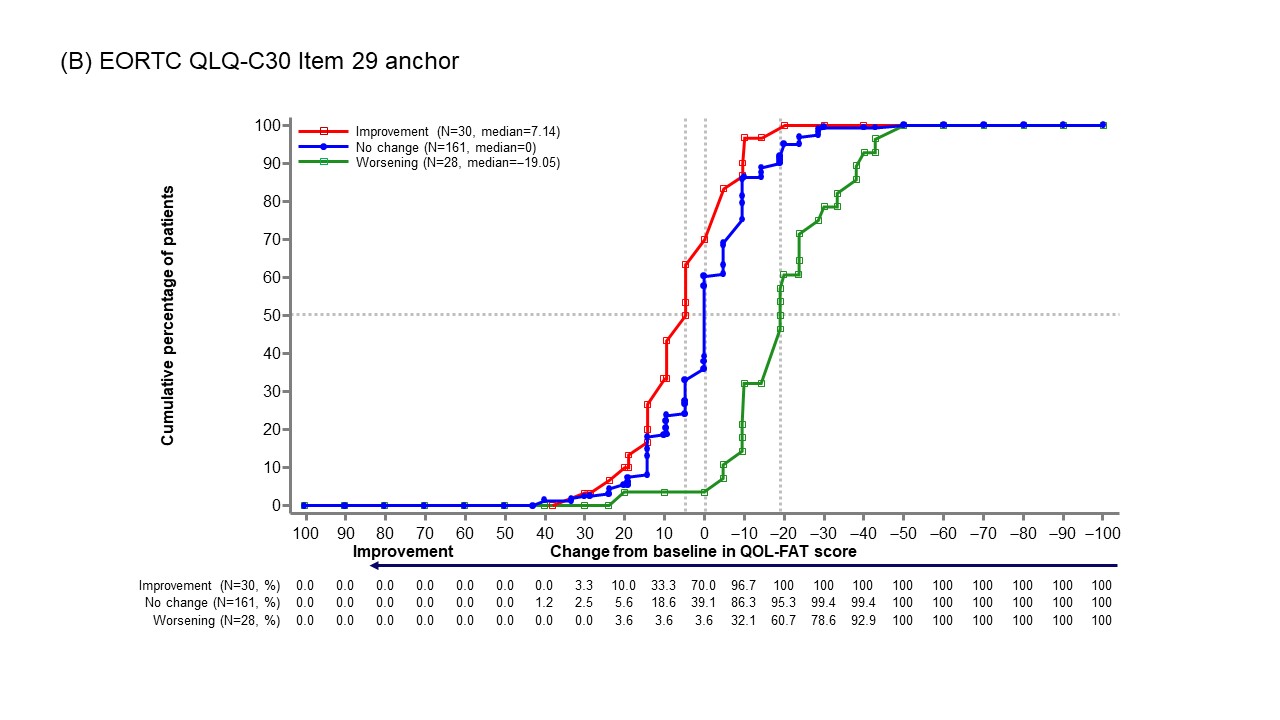
**

eCDF, empirical cumulative distribution function; EORTC, European Organization for Research and Treatment of Cancer; QLQ-C30, Quality of Life Questionnaire-Core 30; QOL-FAT, fatigue.

Data from patients participating in the RevMDS study were excluded from the EORTC QLQ-C30 anchor-based analyses as the EORTC QLQ-C30 was not administered in that study.

## SUPPLEMENTARY FIGURE 5 eCDF of Change from baseline to Week 24 on QOL-MDSS.

**
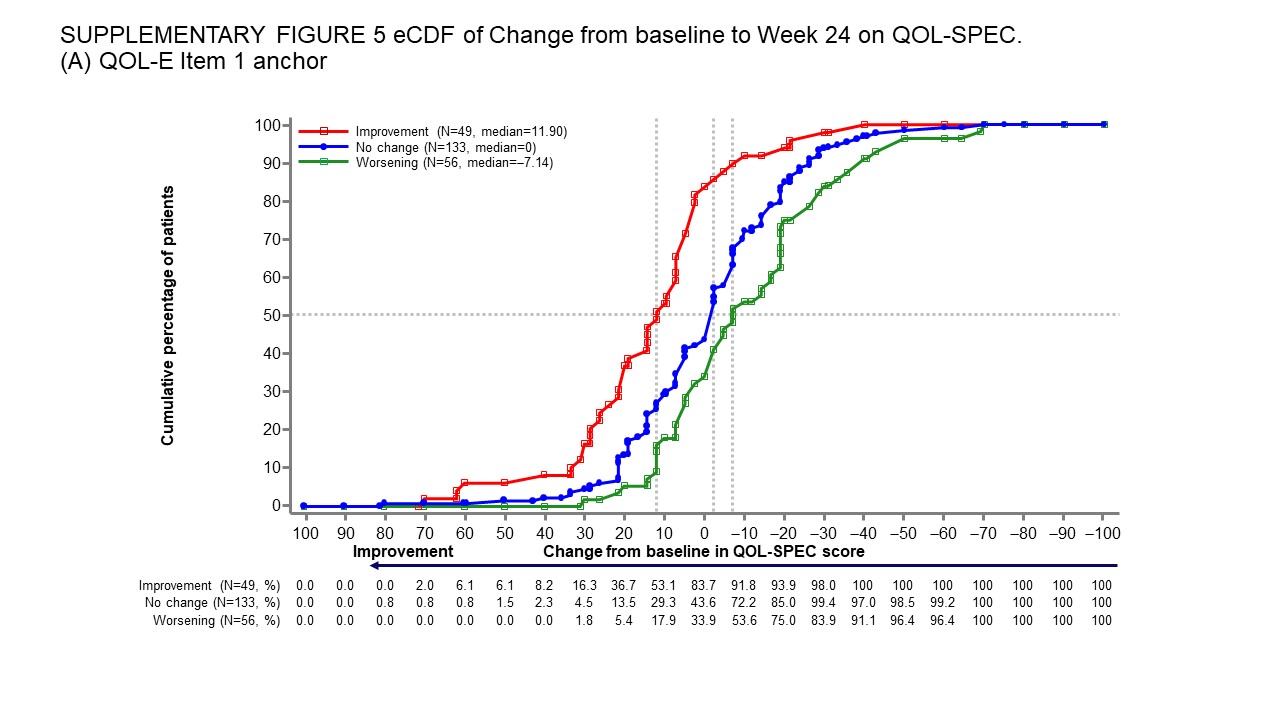
**

**
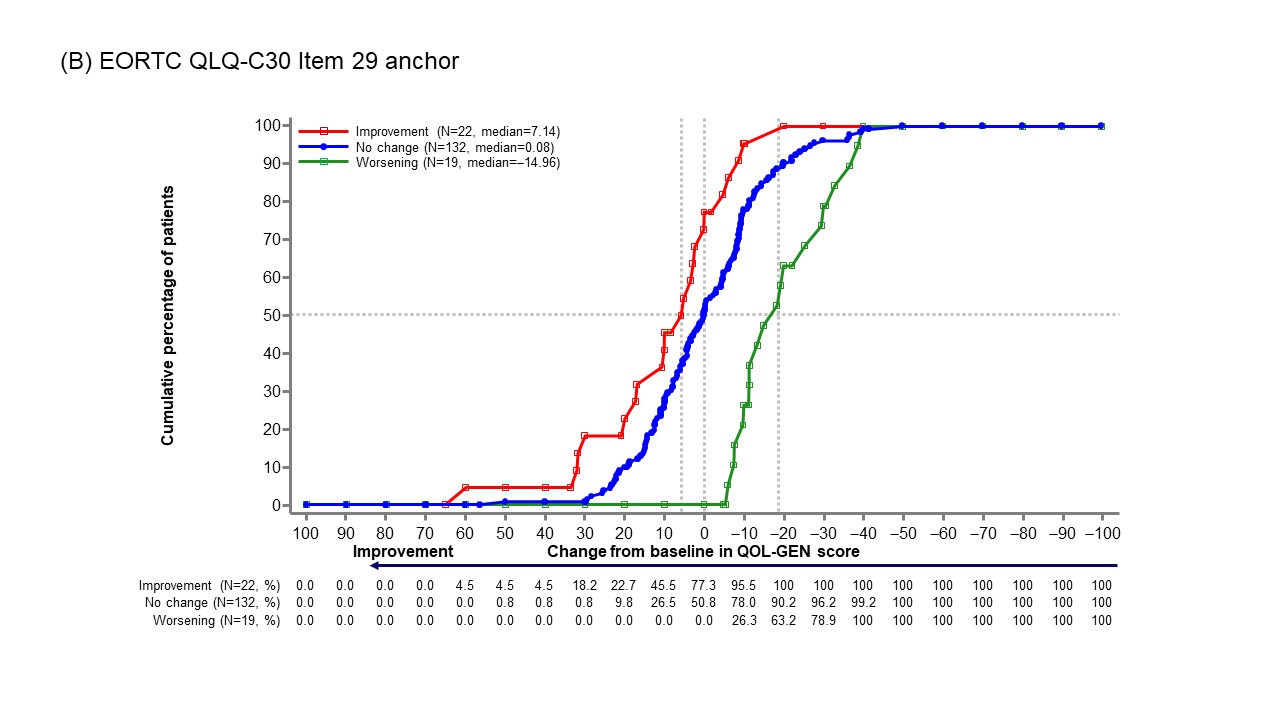
**

eCDF, empirical cumulative distribution function; EORTC, European Organization for Research and Treatment of Cancer; MDS, myelodysplastic neoplasms; QLQ-C30, Quality of Life Questionnaire-Core 30; QOL-MDSS, MDS-specific disturbances.

Data from patients participating in the RevMDS study were excluded from the EORTC QLQ-C30 anchor-based analyses as the EORTC QLQ-C30 was not administered in that study.

SUPPLEMENTARY FIGURE 6 eCDF of change from baseline to Week 24 on QOL-GEN.


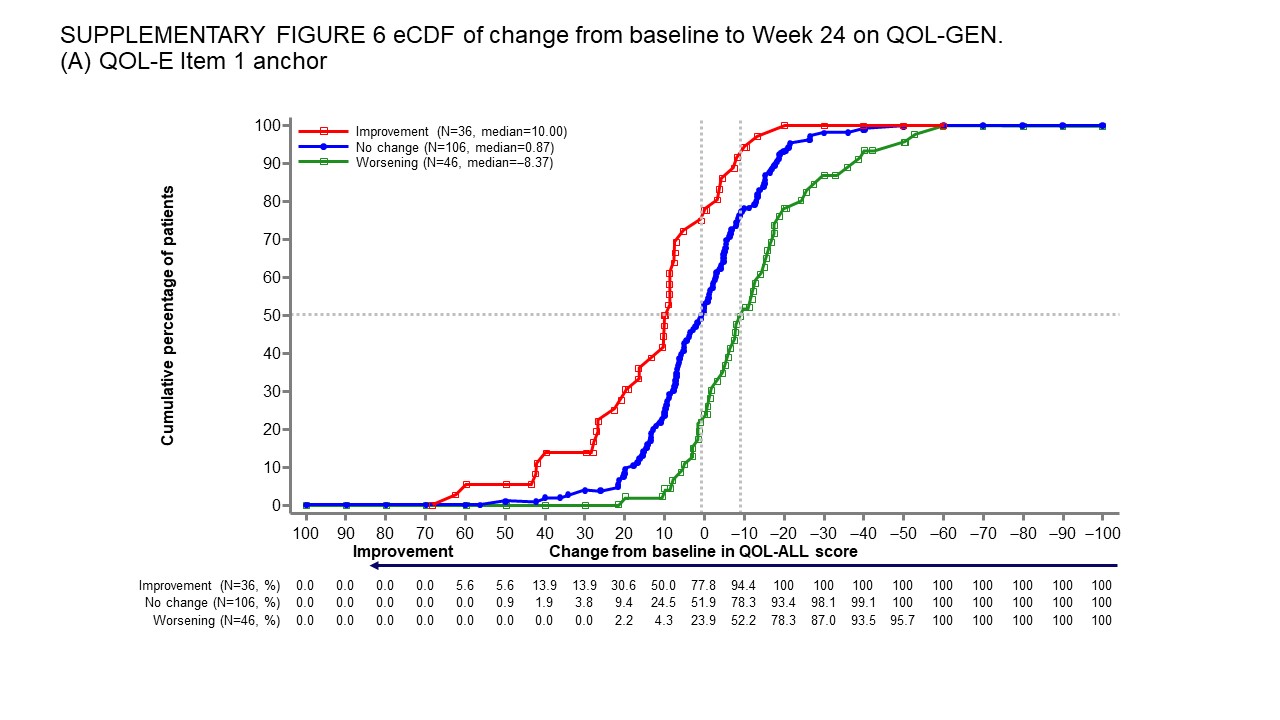

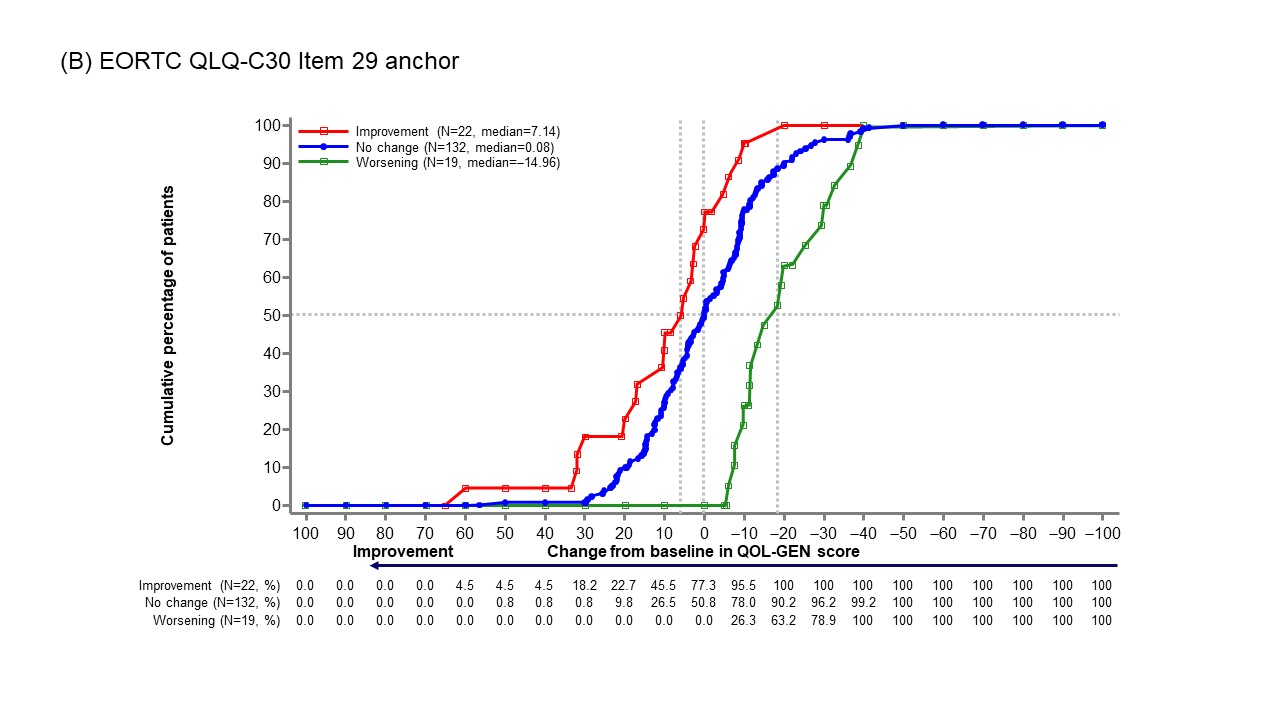


eCDF, empirical cumulative distribution function; EORTC, European Organization for Research and Treatment of Cancer; MDS, myelodysplastic neoplasms; QLQ-C30, Quality of Life Questionnaire-Core 30; QOL-GEN, calculated by taking the mean of all domains except for QOL-MDSS; QOL-MDSS, MDS-specific disturbances.

Data from patients participating in the RevMDS study were excluded from the EORTC QLQ-C30 anchor-based analyses as the EORTC QLQ-C30 was not administered in that study.

## **SUPPLEMENTARY FIGURE 7 eCDF of change from baseline to Week 24 on QOL-ALL.**


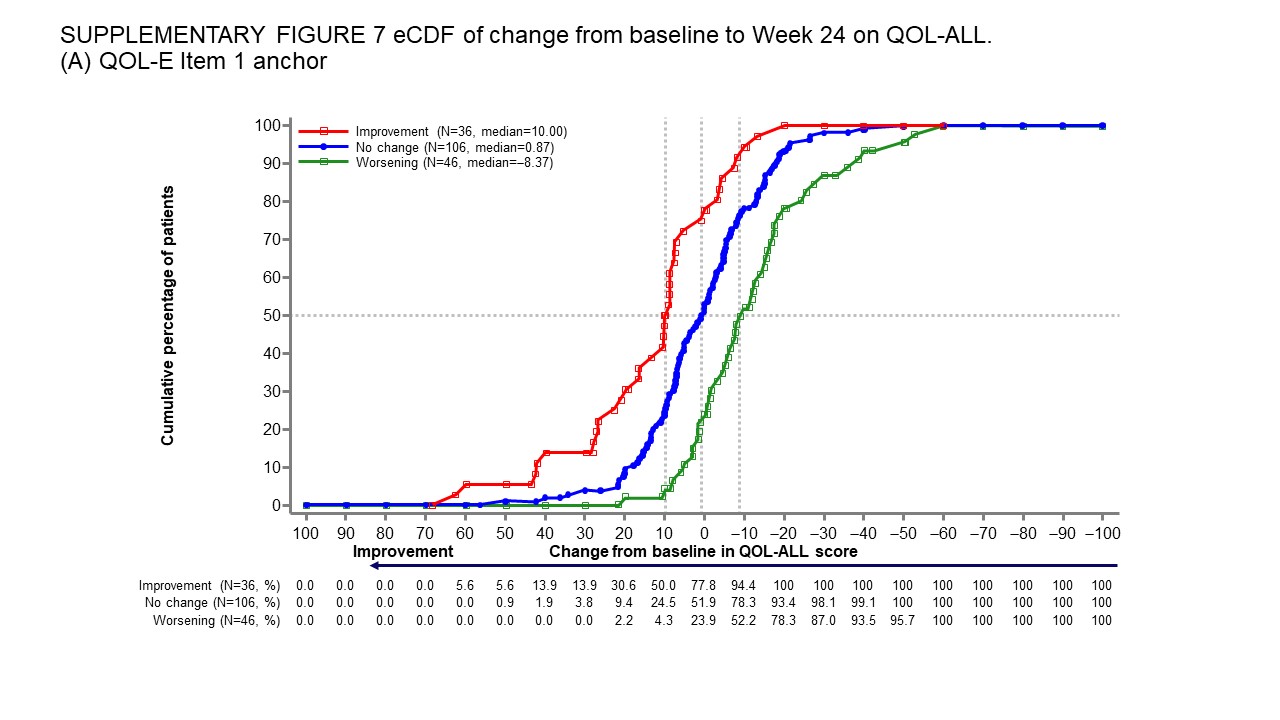

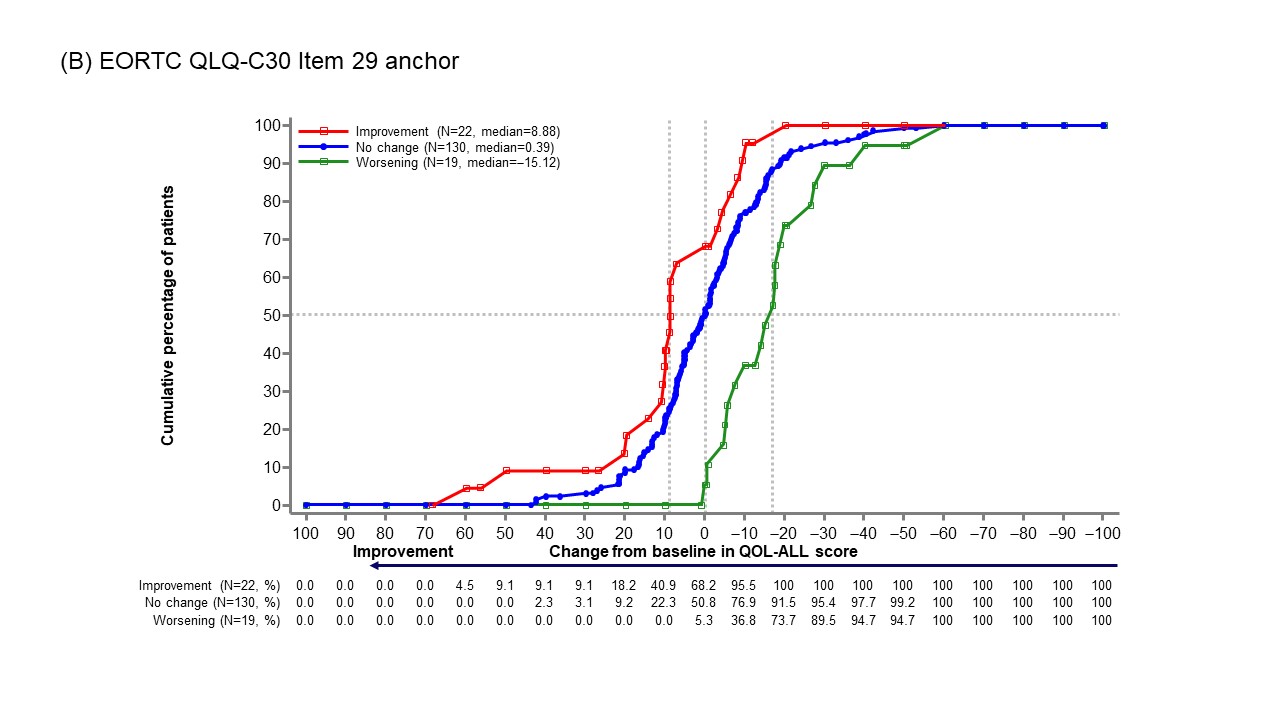


eCDF, empirical cumulative distribution function; EORTC, European Organization for Research and Treatment of Cancer; MDS, myelodysplastic neoplasms; QLQ-C30, Quality of Life Questionnaire-Core 30; QOL-ALL, calculated by taking the mean of QOL-GEN and QOL-MDSS; QOL-GEN, calculated by taking the mean of all domains except for QOL-MDSS; QOL-MDSS, MDS-specific disturbances.

Data from patients participating in the RevMDS study were excluded from the EORTC QLQ-C30 anchor-based analyses as the EORTC QLQ-C30 was not administered in that study.

## SUPPLEMENTARY FIGURE 8 eCDF of change from baseline to Week 24 on QOL-TOI.

**
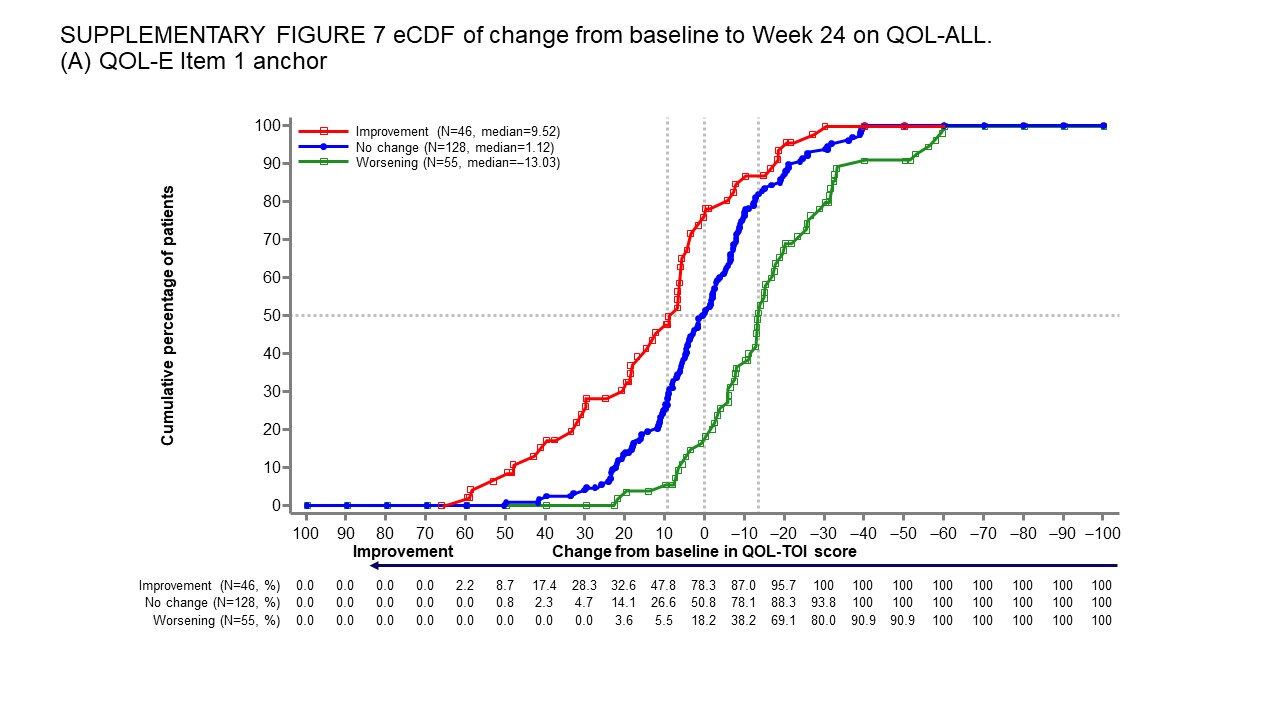
**

**
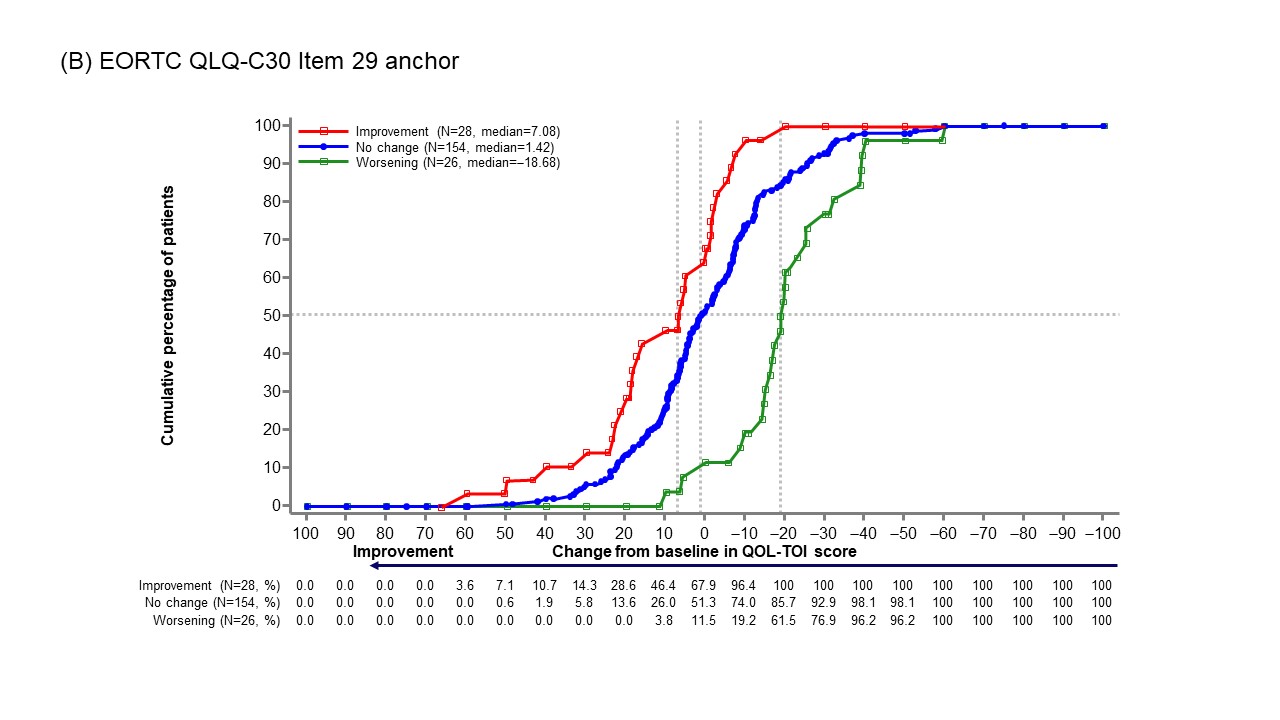
**

eCDF, empirical cumulative distribution function; EORTC, European Organization for Research and Treatment of Cancer; MDS, myelodysplastic neoplasms; QLQ-C30, Quality of Life Questionnaire-Core 30; QOL-FIS, physical well-being; QOL-FUN, functional well-being; QOL-MDSS, MDS-specific disturbances; QOL-TOI, treatment outcome index calculated by taking the mean of QOL-FIS, QOL-FUN, and QOL-MDSS.

Data from patients participating in the RevMDS study were excluded from the EORTC QLQ-C30 anchor-based analyses as the EORTC QLQ-C30 was not administered in that study.

# References

1. Fenaux P, Platzbecker U, Mufti GJ, Garcia-Manero G, Buckstein R, Santini V, et al. Luspatercept in patients with lower-risk myelodysplastic syndromes. *N Engl J Med.* (2020) 382:140-51. doi: 10.1056/NEJMoa1908892
2. Oliva EN, Platzbecker U, Garcia-Manero G, Mufti GJ, Santini V, Sekeres MA, et al. Health-related quality of life outcomes in patients with myelodysplastic syndromes with ring sideroblasts treated with luspatercept in the MEDALIST phase 3 trial. *J Clin Med.* (2022) 11, 27. doi.org/10.3390/ jcm11010027
3. Oliva EN, Nobile F, Alimena G, Specchia G, Danova M, Rovati B, et al. Darbepoetin alfa for the treatment of anemia associated with myelodysplastic syndromes: efficacy and quality of life. *Leuk Lymphoma.* (2010) 51:1007-14. doi: 10.3109/10428191003728610
4. Oliva EN, Riva M, Niscola P, Santini V, Breccia M, Giai V, et al. Eltrombopag for low-risk myelodysplastic syndromes with thrombocytopenia: interim results of a phase ii, randomized, placebo-controlled clinical trial (EQoL-MDS). *J Clin Oncol.* (2023) 41:4486-96. doi: 10.1200/JCO.22.02699
5. Oliva EN, Latagliata R, Lagana C, Breccia M, Galimberti S, Morabito F, et al. Lenalidomide in International Prognostic Scoring System Low and Intermediate-1 risk myelodysplastic syndromes with del(5q): an Italian phase II trial of health-related quality of life, safety and efficacy. *Leuk Lymphoma.* (2013) 54:2458-65. doi: 10.3109/10428194.2013.778406
6. Bedard G, Zeng L, Zhang L, Lauzon N, Holden L, Tsao M, et al. Minimal important differences in the EORTC QLQ-C30 in patients with advanced cancer. *Asia Pac J Clin Oncol.* (2014) 10:109-17. doi: 10.1111/ajco.12070
7. Platzbecker U, Fenaux P, Adès L, Giagounidis A, Santini V, van de Loosdrecht AA, et al. Proposals for revised IWG 2018 hematological response criteria in patients with MDS included in clinical trials. *Blood.* (2019) 133:1020-30. doi: 10.1182/blood-2018-06-857102
